# Octupole corner state in a three-dimensional topological circuit

**DOI:** 10.1038/s41377-020-00381-w

**Published:** 2020-08-19

**Authors:** Shuo Liu, Shaojie Ma, Qian Zhang, Lei Zhang, Cheng Yang, Oubo You, Wenlong Gao, Yuanjiang Xiang, Tie Jun Cui, Shuang Zhang

**Affiliations:** 1 Key Laboratory of Optoelectronic Devices and Systems of Ministry of Education and Guangdong Province, College of Optoelectronic Engineering, Shenzhen University, 518060 Shenzhen, China; 2 School of Physics and Astronomy, University of Birmingham, Birmingham, B15 2TT UK; 3 State Key Laboratory of Millimeter Waves, Southeast University, 210096 Nanjing, China; 4 School of Physics and Electronics, Hunan University, 410082 Changsha, China

**Keywords:** Photonic devices, Metamaterials

## Abstract

Higher-order topological insulators (HOTIs) represent a new family of topological materials featuring quantized bulk polarizations and zero-dimensional corner states. In recent years, zero-dimensional corner states have been demonstrated in two-dimensional systems in the form of quadrupole modes or dipole modes. Due to the challenges in designing and constructing three-dimensional systems, octupole corner modes in 3D have not been observed. In this work, we experimentally investigate octupole topological phases in a three-dimensional electrical circuit, which can be viewed as a cubic lattice version of the Hofstadter model with a *π*-flux threading each plaquette. We experimentally observe in our higher-order topological circuit a 0D corner state manifested as a localized impedance peak. The observed corner state in the electrical circuit is induced by the octupole moment of the bulk circuit and is topologically protected by anticommuting spatial symmetries of the circuit lattice. Our work provides a platform for investigating higher-order topological effects in three-dimensional electrical circuits.

## Viewing topological effects in a 3D electrical circuit

An electrical circuit mimicking the characteristics of a topological insulator (TI) allows the experimental realization of exotic quantum conducting states. TIs, which have applications in the burgeoning fields of spintronics and quantum computing, act as conductors in their bulk but have two-dimensional conducting states on their surfaces. Shuang Zhang at the University of Birmingham, UK and co-workers are using electrical components to mimic the atoms in higher-order TIs, which feature zero-dimensional corner states topologically protected by three anticommuting reflection symmetries of the bulk lattice. The researchers built a 3D topological circuit comprising a cubic lattice of capacitors and inductors and observed a localized peak in impedance spectrum caused by an ‘octupole’ zero-dimensional corner state. Their circuit, which imitates the famous Hofstadter butterfly model of interacting electrons, opens up a new platform for investigating higher-order topological effects.

## Introduction

Topological phases of matter possessing quantized invariants have attracted growing interest not only in the field of condensed matter physics but also in classical systems, such as photonics and acoustics, and have shown great potential in lasing^1–3^, quantum computing^4,5^, and robust signal transmission in optical^6–8^, acoustic^9,10^, and mechanical^11,12^ systems. While most of the research interests for topological insulators have focused on protected nontrivial localized modes one dimension lower than the bulk material, the recent emergence of higher-order topological insulators (HOTIs) shows the possibility of further dimensional reduction of the edge states^13–18^. These quantized higher-order multipole moments are localized at the intersection of the edges of a square (two-dimensional (2D), quadrupole moment) or cubic (three-dimensional (3D), octupole moment) lattice and are protected by spatial symmetries. Thus far, HOTIs are mostly studied in 2D systems that host a quadrupole corner state, such as 2D microwave circuits^19^, low-frequency electrical circuits^20^, photonic crystals^21–25^, mechanical systems^26^, and acoustic systems^27^. The 3D topological corner mode has been demonstrated very recently^28–31^; however, some of these modes result from the nontrivial Zak phase of 3D bulk states^28^, which is of a very different origin than octupole modes.

Here we experimentally observe the third-order topological corner state induced by the octupole moment in a 3D electrical circuit. Electrical circuits have recently emerged as a new potential platform for exploring topological models, such as the Haldane model and magnetic dipoles^32^, spin Hall effect^33,34^, one-dimensional (1D) Su–Schrieffer–Heeger (SSH) model^35,36^, and Weyl states and Fermi arc surface states^37–39^. A few works have been reported on the observation of higher-order topological states in electrical circuits, including 2D square circuit lattices^20^, breathing Kagome and pyrochlore circuit lattices^40^, and anisotropic honeycomb and diamond circuit lattices^41^. However, all these works were limited to either 2D lattices hosting quadrupole corner states or only simulations. In this work, we experimentally demonstrate a 3D topological circuit with a quantized octupole moment, which is manifested by a corner state located at one of the cubic corners. The observed higher-order corner state originates from the octupole moment of the bulk circuit, which is protected by the anticommuting reflection symmetries along all three axes of the bulk circuit. It is noted that the circuit modelling and measurement in this work are derived from the two foundation works, refs. ^20,38^.

## Results

### Bulk circuit diagram and symmetries

**Fig. 1:**
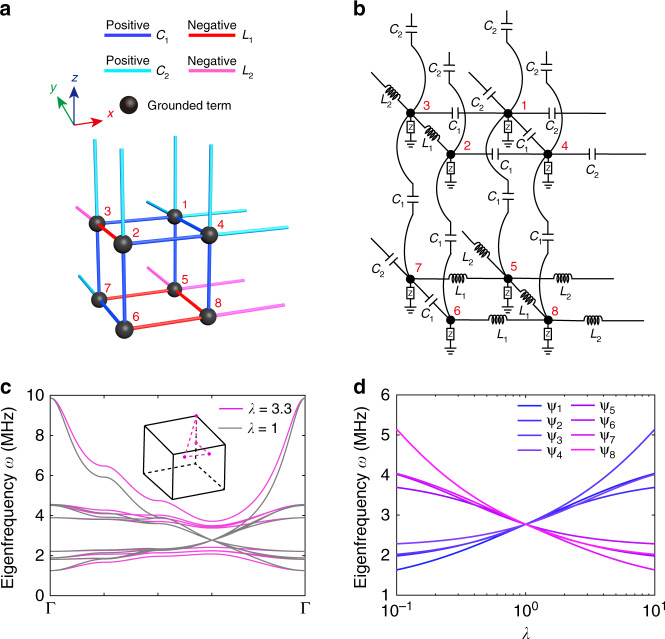
Unit cell and bulk band structure of the higher-order topological circuit hosting the octupole corner state. **a** Theoretical model. Numbers indicate the basis for the circuit Laplacian matrices and Pauli matrices. **b** Circuit diagram of the unit cell. The grounded terms ‘Z’ are given in Supplementary Tables S2–S6. **c** Band structure of the bulk circuit for *λ* = 3.3 (pink) and *λ* = 1 (grey) along high symmetry lines, as indicated in the inset. **d** Eigenfrequencies of the eight states at the *R* point as *λ* is swept from 0.1 to 10

Figure 1a illustrates the theoretical model of the bulk unit cell for the 3D topological circuit possessing an octupole moment, which consists of a cubic lattice with bond dimerization in the *x*, *y*, and *z* directions. Each plaquette (the minimum loop in each plane) in the *xoy*, *yoz*, and *xoz* planes contains one coupling that has the opposite sign to the other three, making the model a cubic lattice version of the Hofstadter model with *π*-flux per plaquette^42^. This is critical for generating a synthetic magnetic *π*-flux threading the plaquette that gives the octupole corner state in the finite-sized system.

The theoretical model in Fig. 1a can be converted into an electrical circuit by implementing four different couplings with two sets of capacitors and inductors (*C*_1_, *L*_1_) and (*C*_2_, *L*_2_), as illustrated by the circuit diagram in Fig. 1b. The latter set is related to the first set by a parameter *λ* through *C*_2_ = *λC*_1_ and *L*_2_ = *L*_1_*/λ* to ensure that both pairs have the same resonant frequency $\omega _0 = 1/\sqrt {L_1C_1} = 1/\sqrt {L_2C_2}$.

The response of an electrical circuit can be explicitly described by a circuit Laplacian ***J***(*ω*), which relates the total input current *I*_*a*_ flowing out of node *a* to the contribution of all other voltages *V*_*b*_ across nodes *a* and *b*,

1 $${\boldsymbol{J}}\left( \omega \right) = \left( {i\omega {\boldsymbol{C}} + \frac{1}{{i\omega }}{\boldsymbol{W}}} \right)$$

where ***C*** and ***W*** are the capacitance and inverse inductance matrices, respectively. The diagonal and off-diagonal terms represent the self-admittance of a certain node and mutual admittance between two nodes, respectively. ***J***(*ω*) is purely imaginary when there are only capacitors and inductors and becomes complex when resistors are present. Because inductors function as the negative counterpart of capacitors at the resonant frequency, that is, $1/C \equiv - \omega _0^2L$, a grounded element composed of inductors and/or capacitors is attached to each node (indicated by the letter ‘Z’ in Fig. 1b) to neutralize the admittance of each node to maintain zero admittance for all the diagonal elements at the resonant frequency *ω*_0_ in the Laplacian matrix (Supplementary Table S1). More details of the modelling, characterization and measurement of the topological circuit can be found in refs. ^20,38^.

The circuit Laplacian ***J***_*λ*_(*ω*, ***q***) of the bulk circuit, which consists of periodically repeating unit cells, can be obtained by substituting the matrices ***C*** and ***W*** given in Supplementary Eqs. (S4) and (S5) into Eq. (2). Here, *q* is the quasi-wave vector linking the voltages on unit cells *n* and *n* + 1 as *V*_*n*+1_ = *V*_*n*_*e*^*iq*^. In the quantum model, a higher-order corner mode emerges at zero energy due to the zero onsite terms of its bulk Hamiltonian^13^. For the circuit analogue with nonzero diagonal elements in the circuit Laplacian, the corner mode is expected to appear at the midgap frequency *ω*_0_. The topological circuit is a dispersive system in which all terms (onsite and coupling) are highly dependent on the frequency; here we only focus on the properties of the bulk circuit Laplacian ***J***_*λ*_(*ω*, ***q***) at *ω* = *ω*_0_,

2 $${\boldsymbol{J}}_\lambda \left( {\omega _0,{\boldsymbol{q}}} \right) = i\sqrt {\frac{{C_1}}{{L_1}}} \left[ {\lambda \sin q_y{\Gamma}_1^\prime + \left( {1 + \lambda \cos q_y} \right){\Gamma}_2^\prime + \lambda \sin q_x{\Gamma}_3^\prime + \left( {1 + \lambda \cos q_x} \right){\Gamma}_4^\prime + \lambda \sin q_z{\Gamma}_5^\prime + \left( {1 + \lambda \cos q_z} \right){\Gamma}_6^\prime } \right]$$

where ${\Gamma}_2^\prime = \xi _3 \otimes {\Gamma}_0$, ${\Gamma}_1^\prime = \xi _3 \otimes {\Gamma}_1$, ${\Gamma}_2^\prime = \xi _3 \otimes {\Gamma}_2$, ${\Gamma}_3^\prime = \xi _3 \otimes {\Gamma}_3$, ${\Gamma}_4^\prime = \xi _3 \otimes {\Gamma}_4$,${\Gamma}_5^\prime = \xi _2 \otimes {\mathrm{I}}_{4 \times 4}$, ${\Gamma}_6^\prime = i{\Gamma}_0^\prime {\Gamma}_1^\prime {\Gamma}_2^\prime {\Gamma}_3^\prime {\Gamma}_4^\prime {\Gamma}_5^\prime$, ${\Gamma}_1 = \tau _2 \otimes \sigma _1$, ${\Gamma}_2 = \tau _2 \otimes \sigma _2$, ${\Gamma}_3 = \tau _2 \otimes \sigma _3$, ${\Gamma}_4 = \tau _1 \otimes \sigma _0$, and *τ*_*v*_, *σ*_*v*_, and *ζ*_*v*_ are Pauli matrices corresponding to the internal degrees of freedom within a unit cell, as illustrated by the node indices 1–8 in Fig. 1a, with *v* = 0, 1, 2, 3. The circuit Laplacian ***J***_*λ*_(*ω*_0_, ***q***) in Eq. (3) takes a similar form (up to a factor of *i*) as the bulk Hamiltonian of the model with a quantized octupole moment in ref. ^13^.

Extending from the 2D case with a quadrupole corner state^20^, we remark that the quantized octupole moment in our 3D topological circuit is protected by the presence of all three reflection symmetries, $\hat m_{\mathrm{x}} = \xi _0 \otimes \tau _1 \otimes \sigma _3$, $\hat m_y = \xi _0 \otimes \tau _1 \otimes \sigma _1$, and $\hat m_z = \tau _1 \otimes \sigma _3 \otimes \xi _0$, and chiral symmetry $C = \tau _3 \otimes \sigma _3 \otimes \xi _0$. These symmetry matrices apply to the 8 × 8 circuit Laplacian ***J***_*λ*_(*ω*_0_, ***q***) in momentum space in the following way:

3 $$\hat m_x{\boldsymbol{J}}_\lambda \left( {\omega _0,{\it{q}}_x,{\it{q}}_y,{\it{q}}_z} \right)\hat m^\dagger _x = {\boldsymbol{J}}_\lambda \left( {\omega _0, - {\it{q}}_x,{\it{q}}_y,{\it{q}}_z} \right)$$

4 $$\hat m_y{\boldsymbol{J}}_\lambda \left( {\omega _0,{\it{q}}_x,{\it{q}}_y,{\it{q}}_z} \right)\hat m^\dagger _y = {\boldsymbol{J}}_\lambda \left( {\omega _0,{\it{q}}_x, - {\it{q}}_y,{\it{q}}_z} \right)$$

5 $$\hat m_z{\boldsymbol{J}}_\lambda \left( {\omega _0,{\it{q}}_x,{\it{q}}_y,{\it{q}}_z} \right)\hat m^\dagger _z = {\boldsymbol{J}}_\lambda \left( {\omega _0,{\it{q}}_x,{\it{q}}_y, - {\it{q}}_z} \right)$$

6 $$C{\boldsymbol{J}}_\lambda \left( {\omega _0,{\it{q}}_x,{\it{q}}_y,{\it{q}}_z} \right)C^\dagger = - {\boldsymbol{J}}_\lambda \left( {\omega _0,{\it{q}}_x,{\it{q}}_y,{\it{q}}_z} \right)$$

It is noted from the bulk circuit unit cell that the system does not have an exact reflection symmetry in the *x*- and *z*-directions. It is the effective magnetic fluxes *B*_*x*_ = *B*_*y*_ = *B*_*z*_ = *π*, not the vector potentials _*Ax*_, *A*_*y*_, and *A*_*z*_, that are invariant under the three reflection symmetries $\hat m_x$, $\hat m_y$, *and* $\hat m_z$. Hence, the three reflection symmetries and the chiral symmetry operators in Eqs. (3)–(6) have been fixed by a gauge for application to our 3D circuit with a *π*-flux magnetic field in all three directions. Therefore, the three gauge-fixed reflection symmetries anticommute with each other, that is, $\hat m_x\hat m_y + \hat m_y\hat m_x = 0$,$\hat m_y\hat m_z + \hat m_z\hat m_y = 0$, and $\hat m_x\hat m_z + \hat m_z\hat m_x = 0$. Such anticommutation among the three reflection symmetries is key to achieving a nontrivial topological octupole corner mode. The 3D circuit also respects the three rotational symmetries $\hat C_{4x}$, $\hat C_{4y}$, and $\hat C_{4z}$ along the three axes *x*, *y*, and *z*, respectively, and the three mirror symmetries $\hat m_{xz} = \hat C_{4y}\hat m_x$, $\hat m_{yz} = \hat C_{4x}\hat m_y$, and $\hat m_{xy} = \hat C_{4z}\hat m_x$. Supplementary Eqs. (S12)–(S17) give the matrix representations of the rotational symmetry operators and illustrate how they apply to the 8 × 8 circuit Laplacian ***J***_*λ*_(*ω*_0_, ***q***).

However, when relating a Hamiltonian in an electronic system to a circuit Laplacian, one should keep in mind that the circuit Laplacian itself is dependent on the frequency, and therefore, it does not directly give the eigenfrequency of the system in the same way as the Hamiltonian does in quantum and photonic systems. There are a number of methods for calculating the eigenfrequencies of the circuit from the circuit Laplacian, as detailed in Supplementary Note S2. As the circuit we considered here is composed of only linear elements, i.e. capacitors and inductors, we can calculate the eigenfrequency of the circuit from the dynamical matrix ***D***(***k***) = ***C***^−1/2^(***k***)***W***(***k***)***C***^−1/2^(***k***)^20,36^, with ***C*** and ***W*** given in Supplementary Eqs. (S4) and (S5). In Fig. 1c, we show the eigenfrequencies of the bulk circuit calculated along the high symmetry lines with parameters of *C*_1_ = 1 nF, *L*_1_ = 3.3 μH, and *λ* = 3.3 (pink curves), which exhibit a complete nontrivial bandgap from 2.4 to 3.8 MHz. Similar to the tight-binding model, the band structure undergoes a phase transition at *λ* = 1 with band closing at the *R* point (*π*, *π*, *π*) (Fig. 1c, grey curves). The topological phase transition can also be observed in Fig. 1d, where the eight eigenfrequencies at the *R* point are plotted as *λ* is swept from 0.1 to 10. The bandgap closes and reopens as *λ* crosses one, with the eigenfrequencies of the eight eigenmodes Ψ_1_–Ψ_8_ crossing *ω*_0_, which is a necessary condition for the presence of any topological state.

### Finite circuit with an octupole corner state

**Fig. 2:**
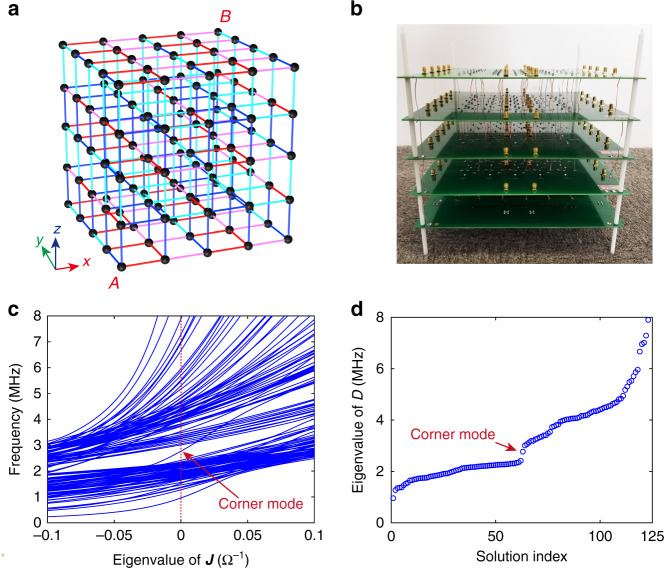
Structure and band properties of the finite circuit containing 2.5 × 2.5 × 2.5 unit cells. **a** Circuit diagram and **b** fabricated sample of the 3D circuit. The coloured lines in the circuit diagram share the same representation as those in Fig. 1a. **c** Eigenvalue of ***J***(*ω*) of the finite circuit as the frequency varies from 0 to 8 MHz. The isolated curve crosses zero admittance at the corner mode frequency 2.77 MHz. This plot has been rotated by 90° to enable a better comparison with the sorted eigenfrequency plot in **d**. **d** Sorted eigenfrequencies of the finite circuit. The isolated mode in the bandgap is the nontrivial octupole corner mode

To observe the topologically protected corner mode in the 3D circuit, we construct a finite-sized circuit, as shown in Fig. 2a, with 2.5 × 2.5 × 2.5 unit cells (5 × 5 × 5 nodes). The diagonal elements of the finite circuit Laplacian matrix should vanish at *ω*_0_ to obey chiral symmetry *C*. The detailed grounded terms for each node are provided in Supplementary Tables S2–S6.

There exists a one-to-one mapping between the eigenvalue spectrum of ***J***(*ω*) and that of the dynamic matrix ***D***, that is, a spectrally isolated eigenfrequency *ω*_0_ of ***D*** corresponds to a spectrally isolated zero eigenvalue of ***J***(*ω*_0_). Figure 2c, d presents the eigenvalue of ***J***(*ω*) and band structure of the system, respectively. An isolated midgap mode (octupole corner state) located at 2.77 MHz can be clearly identified from the band diagram in Fig. 2d. The frequencies where the admittance *j*_*n*_(*ω*) (Fig. 2c, red dashed line) crosses zero correspond exactly to the eigenfrequencies of the circuit (Fig. 2d). The mathematical relation between ***J***(*ω*) and ***D*** implies that the bulk topological invariants of the circuit calculated from the eigenstates of ***J***(*ω*) and ***D*** should be mathematically equivalent.

The nontrivial topological feature of a topological system is manifested by a topologically robust edge state located at the lattice boundaries. Different from conventional topological insulators in electronic and photonic systems, the nontrivial boundary state in topological circuits is commonly observed through a two-point impedance measurement between node *a* and node *b*, subject to an external excitation current *I*_0_ flowing through them^20,36,38^. According to the definition of *Z*_*ab*_(*ω*) = (*V*_*a*_ − *V*_*b*_*)/I*_0_, one can express it with the inversion of Eq. (1) as

7 $$Z_{ab}\left( \omega \right) = \frac{{V_a - V_b}}{{I_0}} = \mathop {\sum}\limits_n {\frac{{\left| {\psi _{n,a} - \psi _{n,b}} \right|^2}}{{j_n\left( \omega \right)}}}$$

in which Ψ_*n*,*i*_. (*i* = *a* or *b*) and *j*_*n*_(*ω*) are the eigenstates and eigenvalues of ***J***(*ω*), respectively. As the roots of *j*_*n*_(*ω*) correspond to the eigenfrequencies of the circuit, *Z*_*ab*_(*ω*) diverges when the denominator *j*_*n*_(*ω*) crosses zero. Hence, an edge state can be easily identified by a spectrally isolated strong resonant peak from the impedance spectra measured at the circuit boundary. Although the octupole corner states can in principle exist at all eight corners of the cube in the nontrivial phase^13^, in our circuit analogue, only corner A (see Fig. 2a) can host the octupole corner state for the following reasons. First, the boundary termination in our circuit obeys only chiral symmetry *C* and three mirror symmetries $\hat m_{xz}$, $\hat m_{yz}$, and $\hat m_{xy}$, which only allow possible corner states at the two corners labelled A and B (Supplementary Fig. S3). In addition, corners A and B are terminated with different unit cell choices of type I (Fig. 1b) and type II (Supplementary Fig. S3), which correspond to two circuit Laplacian matrices $\tilde J^{\left(\mathrm{I}\right)}_\lambda \left( {\omega _0,k} \right)$ and $\tilde J^{\left( {{\mathrm{II}}} \right)}_{1/\lambda }\left( {\omega _0,k} \right)$, respectively. As unit cell type I is nontrivial for *λ* > 1, only corner A allows the existence of the octupole corner state in our specific model. More details are given in Supplementary Note S3.

Note that the corner mode observed in our 3D topological circuit is induced by the octupole moment of the bulk, which takes a topological invariant of 1/2 and 0 when the system is in the nontrivial and trivial states, respectively. This is confirmed by calculating the topology of the Wannier bands through nested Wilson loops along the *x*, *y*, and *z* axes using the analytical solutions of the eigenstates of the bulk circuit, which is detailed in Supplementary Note S4.

## Experimental results

A 3D circuit cube containing 5 × 5 × 5 nodes (2.5 × 2.5 × 2.5 unit cells) is fabricated to experimentally demonstrate the octupole corner mode (Fig. 2b). The sample consists of five circuit board layers, each fabricated with printed circuit board technology. The five circuit boards are assembled together through copper wires. The impedance spectra are measured using a vector network analyser (Agilent 8753ES) through the predesigned microwave port on the circuit board. The resonant frequency is designed to be 2.77 MHz. The parameter *λ* = 3.3 was deliberately chosen to ensure a clear observation of the corner state in the experiment by considering the choices of commercially available circuit components, as discussed in detail in the ‘Methods and materials’ section.

**Fig. 3:**
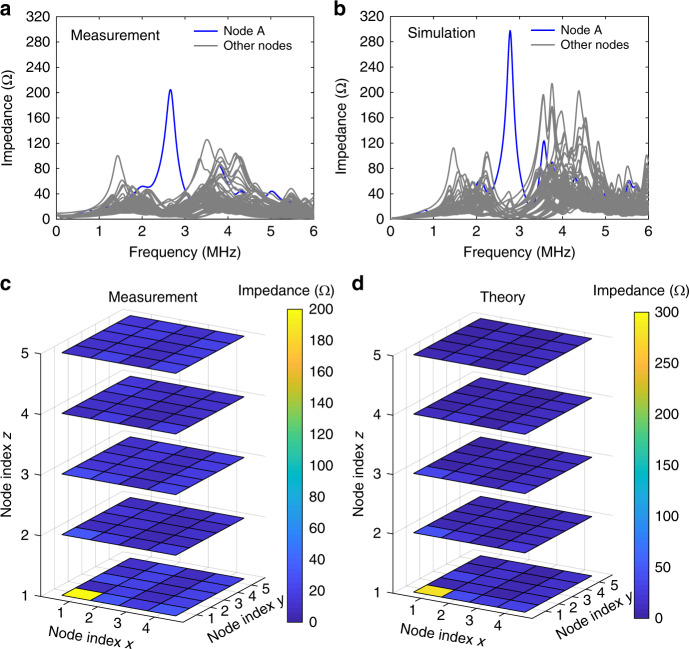
Experimental and theoretically calculated results of the octupole corner state in the finite circuit. **a** Experimentally measured and **b** theoretically calculated impedance spectra at node A. Note that the two-point impedance *Z*_*ab*_ for node A is measured across node A and the next nearest node along the *x*-direction. A *Q*-factor of 40 is set for the inductors in the calculations. **c** Experimentally measured and **d** theoretically calculated impedance distributions of all nodes at the corner mode frequency 2.77 MHz

Figure 3a, b compares the experimental data with the theoretical calculations of the impedance spectra from 30 kHz to 6 MHz, where the blue- and grey-coloured curves represent the impedance spectra measured at corner A and the bulk nodes, respectively. Good agreement is observed between the measured and calculated impedance spectra at four chosen nodes (Supplementary Fig. S1), despite a slight frequency shift of the measured impedance peak due to the parasitic effect of the circuit board layout. The measured impedance peak is slightly lower than the calculation, which is attributed to the lower *Q*-factor of the real circuit components. This influence can be clearly observed in the calculated impedance spectra at corner A with a *Q*-factor from 10 to 80 (Supplementary Fig. S2).

To directly visualize the corner state in the cubic circuit, we plot in Fig. 3c, d the measured and calculated impedance distributions of all nodes at the corner mode frequency 2.77 MHz. Obvious localization of the impedance at corner A is observed in both the measurement and simulation, verifying the existence of a topological corner state. The level of localization of the corner state is determined by the bulk gap. Specifically, the impedance would be less localized at the corner nodes for *λ* close to 1. In contrast to the second-order TI realized with 2D extension of the SSH lattice, where different dimensional (i.e. 1D, 2D) topological boundary states can exist in the same structure^21–23,43^, quadrupole moments and dipole moments are not allowed due to the three anticommuting reflection symmetries.

**Fig. 4:**
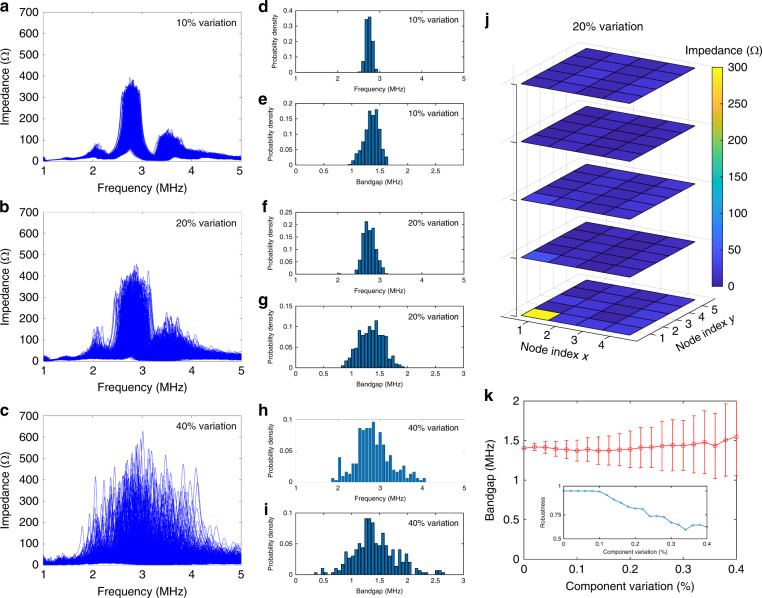
Robustness analysis of the octupole corner state under different component variations. **a**–**c** Five hundred calculated results of the impedance spectra at node A with 10, 20, and 40% circuit component variations, respectively. **d**, **f**, **h** Probability distributions of the corner state frequency with 10, 20, and 40% circuit component variations, respectively. **e**, **g**, **i** Probability distributions of the bandgap of the corner state with 10, 20, and 40% circuit component variations, respectively. **j** Theoretically calculated impedance distributions of all nodes at the corner mode frequency with circuit component variations of 20%. **k** Variation in the corner mode bandgap and robustness (inset) as a function of component variation. Each statistical result in the plot is obtained from 500 random cases. A *Q*-factor of 40 is considered in all simulations

Similar to the 1D edge state (2D surface state) in conventional 2D (3D) topological materials, which exhibits excellent immunity against defects and disorder, the zero-dimensional (0D) corner state in our HOTI circuit is also highly robust against certain types of disorder. To confirm this, we deliberately add different levels of variations to the circuit components in the calculation. Figure 4a–c shows the statistics of 500 calculated results of the impedance spectra with circuit component variations of 10, 20, and 40%, respectively. It can be observed that the frequency of the corner mode is distributed over a larger range around the central frequency of 2.77 MHz, and the level of the frequency shift is proportional to the randomness of the component variation. However, the corner state remains almost unaffected even at 20% circuit component variation, as is confirmed by the theoretically calculated impedance distributions in Fig. 4j.

To obtain a statistical view of the frequency shift of the corner mode for the three cases in Fig. 4a–c, we present in Fig. 4d, f, h the probability distributions of the corner mode frequency under 10, 20, and 40% component variation, respectively. We see that the frequency of the corner mode spans a larger range as the component variation increases. The impedance intensity of the corner mode also experiences a similar trend as the corner mode frequency, as shown in Supplementary Fig. S7. We quantitatively evaluate the robustness of the corner mode by its bandgap, defined as the bandwidth formed between the nearest impedance peaks of the bulk modes across the bandgap. A larger bandgap indicates a more robust corner mode. Figure 4e, g, i shows that, as the component variation increases, the bandgap is distributed over a broader range around the central value of 1.38 MHz. It is important to note that all the results in the statistical charts of Fig. 4d–i show prominent corner modes, with the intensity of the corner mode at least twice that of the bulk modes. The other cases in which the intensity is lower than this threshold are considered as the failed cases in which the corner mode is affected by the component variation. The percentage of the non-failed cases among all cases is defined as the robustness of the corner mode, which is 98, 83.8, and 60.2% for component variations of 10, 20, and 40%, respectively. As the component tolerance of commercially available capacitors and inductors has an upper limit of 20% and is typically 5 and 10%, respectively, this gives rise to a good robustness of the octupole corner mode in real experiments. Figure 4k further reveals how the bandgap and robustness of the corner mode varies as a function of component variation. Each statistical result in the plot is obtained from 500 random cases. It is interesting to find that the robustness (inset of Fig. 4k) is maintained at almost 100% (unaffected) for small component variation and starts to linearly decrease as the component variation exceeds 10%. The average corner mode bandgap remains at approximately 1.38 MHz, while its standard deviation gradually increases from 0 to 0.49 MHz as the component variation increases from 0 to 40%.

## Discussion

In this work, we have experimentally demonstrated a higher-order topological circuit that can host an octupole moment manifested by a topologically nontrivial 0D corner state localized at one of the cubic corners. Our circuit can be viewed as the 3D version of the famous Hofstadter model with *π*-flux per plaquette, in which three gauge-fixed reflection symmetries with anticommutation relations play an essential role in the generation of the octupole moment. Our circuit implementation of octupole topological insulators paves the way for future investigations of higher-dimensional topological insulators possessing multipole moments without introducing synthetic dimensions, benefitting from the convenient electrical connections among nodes at arbitrary distances. In addition, the wide choice of active components, such as operational amplifiers, allows dynamic control of the topology and order of the 3D circuit^44,45^, while nonlinear circuit components, such as varactor diodes, could further introduce strong nonlinear effects into the 3D circuit to realize self-induced topological states^46^ and topologically robust propagating solitons^47^. It is interesting to note that if we apply an alternating current excitation across the entire bulk circuit, then most of the electrical energy will be concentrated at the corner node due to the high impedance, which is analogous to the localized electric field distribution at the surface/edge/corner of a photonic topological material. Therefore, we remark that the experimental realization of an octupole corner state in the electrical circuit system serves as a proof-of-concept demonstration and can be viewed as the low-frequency version of the octupole topological insulator in the photonic regime. Note that, during the revision of this manuscript, we noticed that another work has reported on 3D experimental realization of the octupole corner state^48^.

## Methods and materials

### Experimental details

The parameter *λ* = 3.3, which determines the ratio between the capacitors and inductors, was deliberately chosen based on the following considerations. First, *λ* determines the bulk bandgap and consequently the level of localization of the corner state. Hence, it should not be too small to allow clear experimental observation of the corner state in the impedance measurement. However, we should also consider the minimum inductance *L*_1_/(2 + 3*λ*) of the grounded terms, which should not be too close to the parasitic inductance of the circuit layout (several tens of nH). In addition, we also considered the nominal values of the commercial circuit elements to realize all the precise circuit parameters using a single element or serial/shunt combination of two capacitors/inductors. To meet the above requirements, we chose wire-wound inductors in the surface mounted device package from Murata, which offer an average *Q*-factor of >40 at the working frequency of 2.77 MHz. Note that, in the fabricated sample, the capacitors *C*_1_/*C*_2_ between each adjacent layer are welded on the lower layer.

## Supplementary information

SUPPLEMENTAL MATERIAL
