# Supplementary material for: Octupole corner state in a three-dimensional topological circuit
Source: Light Sci Appl. 2020 Aug 19;9:145. doi: 10.1038/s41377-020-00381-w (PMC7438484; doi:10.1038/s41377-020-00381-w)
Supplement: Supplementary file 1 — SUPPLEMENTAL MATERIAL [file 41377_2020_381_MOESM1_ESM.docx]

**Supplementary Information for**

**Octupole corner state in a three-dimensional topological circuit**

Shuo Liu^1,2^, Shaojie Ma^1,2^, Qian Zhang^3^, Lei Zhang^3^, Cheng Yang^3^, Oubo You^1,2^, Wenlong Gao^2^, Yuan Jiang Xiang^4,*^, Tie Jun Cui^3,*^, Shuang Zhang^2,*^

^1^ Key Laboratory of Optoelectronic Devices and Systems of Ministry of Education and Guangdong Province, College of Optoelectronic Engineering, Shenzhen University, Shenzhen 518060, China

^2^ School of Physics and Astronomy, University of Birmingham, Birmingham B15 2TT, United Kingdom

^3^ State Key Laboratory of Millimeter Waves, Southeast University, Nanjing 210096, China

^4^ School of Physics and Electronics, Hunan University, Changsha 410082, China

*Corresponding author

Corresponding authors: S. Zhang: [S.Zhang@bham.ac.uk](mailto:S.Zhang@bham.ac.uk); T. J. Cui: [Tjcui@seu.edu.cn](mailto:Tjcui@seu.edu.cn); Y. J. Xiang: Xiang78@hnu.edu.cn;

**This PDF file includes:**

Supplementary Text Note S1 to Note S4

Supplementary Figs. S1 to S7

Supplementary Table S1 to S6

**Note S1: Bulk circuit Laplacian**

The circuit response of any non-dissipative electrical circuit network can be described by the equation of motion according to the Kirchhoff’s law,

$\frac{d}{dt}I_{a}=C_{ab}\frac{d^{2}}{dt^{2}}V_{b}+W_{ab}V_{b}$ (S1)

where *I_a_* is the total input current flowing out of node *a*, and *V_b_* is the voltage across node *a* and *b*. *C_ab_* and *W_ab_* are the capacitance and conductance across node *a* and node *b*, respectively. When we apply an AC excitation $V\left(t\right)=V\left(0\right)e^{i\omega t}$ to the circuit, Eq. (S1) can be described by the following equation linking the total input current *I_a_* flowing out of node *a* and the voltage *V_b_* across node *a* and *b*,

$I_{a}=\sum_{\text{b}} \left(i\omega C_{ab}+\frac{1}{i\omega}W_{ab}\right)V_{b}=\sum_{b} J_{ab}\left(\omega\right)V_{b}$ (S2)

and it takes the matrix form,

$\text{J}\left(\omega\right)=\left(i\omega\text{C}+\frac{1}{i\omega}\text{W}\right)$ (S3)

where ***J****(ω)* is the circuit Laplacian which possesses the complete information of a circuit. ***C*** and ***W*** are the Laplacian matrices of capacitance and inverse inductance respectively. The diagonal and off-diagonal components representing the self-admittance of a certain node and mutual admittance between two nodes, respectively. ***J***(ω) is purely imaginary when there is only capacitors and inductors, while becomes complex when resistance is present.

For an infinite topological circuit composed of the unit cell in Fig. 2b, matrices ***C***, ***W*** are given as,

$\boldsymbol{C}=\left[ \begin{matrix} \begin{matrix} \begin{matrix} -(3C_{1}+3C_{2}) & 0 \\ 0 & -(2C_{1}+2C_{2}) \end{matrix} & \begin{matrix} C_{1}+C_{2}e^{+iq_{x}} & C_{1}+C_{2}e^{+iq_{y}} \\ 0 & C_{1}+C_{2}e^{-iq_{x}} \end{matrix} \\ \begin{matrix} C_{1}+C_{2}e^{-iq_{x}} & 0 \\ C_{1}+C_{2}e^{-iq_{y}} & C_{1}+C_{2}e^{+iq_{x}} \end{matrix} & \begin{matrix} -(2C_{1}+2C_{2}) & 0 \\ 0 & -(3C_{1}+3C_{2}) \end{matrix} \end{matrix} & \begin{matrix} \begin{matrix} C_{1}+C_{2}e^{+iq_{z}} & 0 \\ 0 & C_{1}+C_{2}e^{+iq_{z}} \end{matrix} & \begin{matrix} 0 & 0 \\ 0 & 0 \end{matrix} \\ \begin{matrix} 0 & 0 \\ 0 & 0 \end{matrix} & \begin{matrix} C_{1}+C_{2}e^{+iq_{z}} & 0 \\ 0 & C_{1}+C_{2}e^{+iq_{z}} \end{matrix} \end{matrix} \\ \begin{matrix} \begin{matrix} C_{1}+C_{2}e^{-iq_{z}} & 0 \\ 0 & C_{1}+C_{2}e^{-iq_{z}} \end{matrix} & \begin{matrix} 0 & 0 \\ 0 & 0 \end{matrix} \\ \begin{matrix} 0 & 0 \\ 0 & 0 \end{matrix} & \begin{matrix} C_{1}+C_{2}e^{-iq_{z}} & 0 \\ 0 & C_{1}+C_{2}e^{-iq_{z}} \end{matrix} \end{matrix} & \begin{matrix} \begin{matrix} -(C_{1}+C_{2}+C_{1g}) & 0 \\ 0 & -(2C_{1}+2C_{2}) \end{matrix} & \begin{matrix} 0 & 0 \\ C_{1}+C_{2}e^{-iq_{y}} & 0 \end{matrix} \\ \begin{matrix} 0 & C_{1}+C_{2}e^{+iq_{y}} \\ 0 & 0 \end{matrix} & \begin{matrix} -(2C_{1}+2C_{2}) & 0 \\ 0 & -(C_{1}+C_{2}+C_{1g}) \end{matrix} \end{matrix} \end{matrix} \right]$ (S4)

$\boldsymbol{W}=\left[ \begin{matrix} \begin{matrix} \begin{matrix} -(1/L_{1g}) & 0 \\ 0 & -(1/L_{1}+1/L_{2}+1/L_{2g}) \end{matrix} & \begin{matrix} 0 & 0 \\ 1/L_{1}+1/L_{2}e^{-iq_{y}} & 0 \end{matrix} \\ \begin{matrix} 0 & 1/L_{1}+1/L_{2}e^{+iq_{y}} \\ 0 & 0 \end{matrix} & \begin{matrix} -(1/L_{1}+1/L_{2}+1/L_{2g}) & 0 \\ 0 & -(1/L_{1g}) \end{matrix} \end{matrix} & \begin{matrix} \begin{matrix} 0 & 0 \\ 0 & 0 \end{matrix} & \begin{matrix} 0 & 0 \\ 0 & 0 \end{matrix} \\ \begin{matrix} 0 & 0 \\ 0 & 0 \end{matrix} & \begin{matrix} 0 & 0 \\ 0 & 0 \end{matrix} \end{matrix} \\ \begin{matrix} \begin{matrix} 0 & 0 \\ 0 & 0 \end{matrix} & \begin{matrix} 0 & 0 \\ 0 & 0 \end{matrix} \\ \begin{matrix} 0 & 0 \\ 0 & 0 \end{matrix} & \begin{matrix} 0 & 0 \\ 0 & 0 \end{matrix} \end{matrix} & \begin{matrix} \begin{matrix} -(2/L_{1}+2/L_{2}) & 0 \\ 0 & -(1/L_{1}+1/L_{2}+1/L_{2g}) \end{matrix} & \begin{matrix} 1/L_{1}+1/L_{2}e^{+iq_{x}} & 1/L_{1}+1/L_{2}e^{+iq_{y}} \\ 0 & 1/L_{1}+1/L_{2}e^{-iq_{x}} \end{matrix} \\ \begin{matrix} 1/L_{1}+1/L_{2}e^{-iq_{x}} & 0 \\ 1/L_{1}+1/L_{2}e^{-iq_{y}} & 1/L_{1}+1/L_{2}e^{+iq_{x}} \end{matrix} & \begin{matrix} -(1/L_{1}+1/L_{2}++1/L_{2g}) & 0 \\ 0 & -(2/L_{1}+2/L_{2}) \end{matrix} \end{matrix} \end{matrix} \right]$ (S5)

in which quasi momentum *q* relates the *n-th* unit cell and *n±1-th* unit cells with $V_{n\pm1}\left(t\right)=V_{n}\left(t\right)\cdot e^{\pm iq}$

**Note S2: Approaches for obtaining eigenfrequencies of topological circuit**

Different from the Hamiltonian in electronics and photonic systems which directly gives the eigenfrequency of the system, the circuit Laplacian itself is dependent on frequency, thus, the eigenvalues of ***J****(ω,q)* do not correspond to the eigenfrequency of the system. There are three different approaches for calculating the eigenfrequencies of a dissipative circuit from the circuit Laplacian.

*Approach Ⅰ:* The first approach is to solve the roots of the determinant of ***J****(ω,q)* (*det(****J****(ω,q))=0*), which may result in considerable computational complexity, and does not have a closed-form solution when the dimension of matrix ***J****(ω,q)* exceeds five.

*Approach Ⅱ:* If we set *I_a_*=0 in the equation of motion in Eq. (S1), the evolution of circuit is then determined only by its eigenfrequencies. Define a new basis *ψ(t)* which is comprised of voltage $V(\mathrm{t})$ and its first derivative ,

(S6)

As ***C***, ***W*** and ***J****(ω,q)* are *N×N* matrices for an *N*-node circuit network, Eq. (S1) can hence be rewritten into *2N* differential equations,

(S7)

where the *2N×2N* circuit Hamiltonian takes the form,

$H\left(q\right)=i\left[\begin{matrix} 0 & \frac{W}{C} \\ -I & 0 \end{matrix}\right]$ (S8)

in which matrices ***C****,* ***W***are given in Supplementary Eqs. (S4)-(S5). It has been proved that the topological invariant of the circuit calculated from both the circuit Laplacian and circuit Hamiltonian are identical [1].

*Approach Ⅲ:* According to the [Kirchhoff's](http://www.baidu.com/link?url=wpq-Wn7YO-rAzlWZvd5qMzfcrnagI-mv4eamhaoBaZqFf4Qlp6WM4ZMNcASTUzIBI1hGqkkrrPae1ZqidoCmYqtlB_qlvMqWf-qt3Ac3OYqIopFXlrfKQjYua2kVGdag) current Law, the total current flowing into, or out of, a node should be zero,

(S9)

where represents the voltage on each node. Considering varies in the form *e^iωt^*, then Eq.(S9) can be simplified as,

(S10)

Eq. (S10) can be written into the following form up to a gauge transformation $D\text{=}C^{-1/2}WC^{-1/2}$***,***

(S11)

Now, the new dynamical matrix ***D*** gives spectrum *ω^2^* as its eigenvalues and  as its new eigenstate.

**Note S3: Symmetry of the 3D topological circuit**

Eqs. (S12)- (S14) describe how the rotational symmetry operators $\hat{C}_{4x}$*,* $\hat{C}_{4y}$*,* $\hat{C}_{4z}$ and the mirror symmetry operators $\hat{m}_{xz}=\hat{C}_{4y}\hat{m}_{x}$*,* $\hat{m}_{yz}=\hat{C}_{4x}\hat{m}_{y}$*,* and $\hat{m}_{xy}=\hat{C}_{4z}\hat{m}_{x}$ function on the circuit Laplacian $J_{\lambda}\left(\omega_{0},q\right)$

$\hat{c}_{4x}J_{\lambda}\left(\omega_{0},q_{x},q_{y},q_{z}\right)\hat{c}^{\dagger}_{4x}=J_{\lambda}\left(\omega_{0},q_{x},q_{z},-q_{y}\right)$ (S12)

$\hat{c}_{4y}J_{\lambda}\left(\omega_{0},q_{x},q_{y},q_{z}\right)\hat{c}^{\dagger}_{4y}=J_{\lambda}\left(\omega_{0},q_{z},q_{y},-q_{x}\right)$ (S13)

$\hat{c}_{4z}J_{\lambda}\left(\omega_{0},q_{x},q_{y},q_{z}\right)\hat{c}^{\dagger}_{4y}=J_{\lambda}\left(\omega_{0},q_{y},-q_{x},q_{z}\right)$ (S14)

where matrix representations of $\hat{C}_{4x}$*,* $\hat{C}_{4y}$*,* $\hat{C}_{4z}$ in our basis are written as,

$\hat{c}_{4x}=\left[\begin{matrix} \begin{matrix} 0 & 0 & 0 & 1 \\ 0 & 0 & 0 & 0 \\ 0 & 1 & 0 & 0 \\ 0 & 0 & 0 & 0 \end{matrix} & \begin{matrix} 0 & 0 & 0 & 0 \\ 0 & -1 & 0 & 0 \\ 0 & 0 & 0 & 0 \\ 0 & 0 & 0 & 1 \end{matrix} \\ \begin{matrix} 1 & 0 & 0 & 0 \\ 0 & 0 & 0 & 0 \\ 0 & 0 & -1 & 0 \\ 0 & 0 & 0 & 0 \end{matrix} & \begin{matrix} 0 & 0 & 0 & 0 \\ 0 & 0 & -1 & 0 \\ 0 & 0 & 0 & 0 \\ -1 & 0 & 0 & 0 \end{matrix} \end{matrix}\right]$ (S15)

$\hat{c}_{4y}=\left[\begin{matrix} \begin{matrix} 0 & 0 & -1 & 0 \\ 0 & 0 & 0 & 0 \\ 0 & 0 & 0 & 0 \\ 0 & 1 & 0 & 0 \end{matrix} & \begin{matrix} 0 & 0 & 0 & 0 \\ 0 & 1 & 0 & 0 \\ 0 & 0 & -1 & 0 \\ 0 & 0 & 0 & 0 \end{matrix} \\ \begin{matrix} -1 & 0 & 0 & 0 \\ 0 & 0 & 0 & 0 \\ 0 & 0 & 0 & 0 \\ 0 & 0 & 0 & 1 \end{matrix} & \begin{matrix} 0 & 0 & 0 & 0 \\ 0 & 0 & 0 & -1 \\ 1 & 0 & 0 & 0 \\ 0 & 0 & 0 & 0 \end{matrix} \end{matrix}\right]$ (S16)

$\hat{c}_{4z}=\left[\begin{matrix} \begin{matrix} 0 & 0 & 1 & 0 \\ 0 & 0 & 0 & 1 \\ 0 & -1 & 0 & 0 \\ 1 & 0 & 0 & 0 \end{matrix} & \begin{matrix} 0 & 0 & 0 & 0 \\ 0 & 0 & 0 & 0 \\ 0 & 0 & 0 & 0 \\ 0 & 0 & 0 & 0 \end{matrix} \\ \begin{matrix} 0 & 0 & 0 & 0 \\ 0 & 0 & 0 & 0 \\ 0 & 0 & 0 & 0 \\ 0 & 0 & 0 & 0 \end{matrix} & \begin{matrix} 0 & 0 & 1 & 0 \\ 0 & 0 & 0 & 1 \\ 0 & -1 & 0 & 0 \\ 1 & 0 & 0 & 0 \end{matrix} \end{matrix}\right]$ (S17)

Eqs. (S18)- (S20) describe how the mirror symmetry operators $\hat{m}_{xz}=\hat{C}_{4y}\hat{m}_{x}$*,* $\hat{m}_{yz}=\hat{C}_{4x}\hat{m}_{y}$*,* and $\hat{m}_{xy}=\hat{C}_{4z}\hat{m}_{x}$ function on the circuit Laplacian $J_{\lambda}\left(\omega_{0},q\right)$

$\hat{m}_{xz}J_{\lambda}\left(\omega_{0},q_{x},q_{y},q_{z}\right)\hat{m}^{\dagger}_{xz}=J_{\lambda}\left(\omega_{0},q_{z},q_{y},q_{x}\right)$ (S18)

$\hat{m}_{yz}J_{\lambda}\left(\omega_{0},q_{x},q_{y},q_{z}\right)\hat{m}^{\dagger}_{yz}=J_{\lambda}\left(\omega_{0},q_{x},q_{z},q_{y}\right)$ (S19)

$\hat{m}_{xy}J_{\lambda}\left(\omega_{0},q_{x},q_{y},q_{z}\right)\hat{m}^{\dagger}_{xy}=J_{\lambda}\left(\omega_{0},q_{y},q_{x},q_{z}\right)$ (S20)

These three *C_4_* rotational symmetries have also been fixed by the gauge, so that they relate to the three reflection symmetries as $\hat{c}_{4x}\hat{m}_{y}-\hat{m}_{z}\hat{c}_{4x}=0$,$\hat{c}_{4y}\hat{m}_{x}-\hat{m}_{z}\hat{c}_{4y}=0$, $\hat{c}_{4z}\hat{m}_{y}-\hat{m}_{x}\hat{c}_{4z}=0$.

The following are the reasons why the corner state is only observed at one corner of our finite 3D circuit. Firstly, the edge termination allows only chiral symmetry and three mirror symmetries for our finite 3D circuit at *ω_0_*. Only the two corners labeled with A and B are protected simultaneously by the three mirror symmetries $\hat{m}_{xz}$*,* $\hat{m}_{yz}$*,* and $\hat{m}_{xy}$, as visualized in Supplementary Fig. S3, in which red hinges represent the points in real space that are protected by each mirror symmetry. Secondly, the unit cell of the topological circuit can be cut in two different ways, resulting in two different choices for the unit cell, as shown in Fig. 1b (type-Ⅰ) and Supplementary Fig. S4 (type-Ⅱ). They correspond to two circuit Laplacian matrices and , respectively, which satisfy . The nontrivial corner state only exists at the corner (corner A) terminated with type-Ⅰ unit cell with λ > 1, because in this condition the corner at the other end of the cube diagonal (corner B) is terminated by the trivial type-Ⅱ unit cell.

**Note S4: Topology of the octupole moment.**

***Procedure for calculating the nested Wilson loop:*** Note that the corner mode observed in our 3D topological circuit is induced by the octupole moment of the bulk, which takes a topological invariant of 1/2 and 0 when the system is in the nontrivial and trivial state, respectively. The process to determine the topology of an octupole moment is illustrated in Fig. S5, which involves a successive calculation of three nested Wilson loops along *q_z_*, *q_y_*, and *q_x_*. The 1^st^-round Wilson loop is obtained by calculating the 4×4 non-abelian Wilson loop along *q_z_* using the four positive eigenvectors of$J_{\lambda}\left(\omega_{0},q\right)$, which are four-fold degenerate in the entire 3D BZ, as presented in Fig. S5a. The 1^st^-round Wilson loop gives two two-fold degenerate Wannier bands, with positive and negative Wannier eigenvalues $\nu_{z}^{\pm,j}\left(k\right)$ shown in Fig. S5b. A new Wannier state $|w_{z,k}^{+,j}\rangle\text{=}\sum_{n=1}^{N_{occ}} |u_{k}^{n}\rangle\left[v_{z,k}^{+,j}\right]^{n}$ is constructed and used for the 2^nd^-round 2×2 non-abelian Wilson loop calculated along *q_y_*, which gives two separate Wannier bands, with positive and negative Wannier eigenvalues $\eta_{y}^{\pm}\left(k\right)$ shown in Fig. S5c. The final step is to calculate the 3^rd^-round Wilson loop $\tilde{W}_{x,k}^{\pm}$ using the constructed Wannier states $|w_{y,k}^{\pm}\rangle\text{=}\sum_{n=1}^{N_{occ}} |u_{k}^{n}\rangle\left[\eta_{y,k}^{\pm}\right]^{n}$. Since the Wannier values $\nu_{y}^{j,\pm}\left(k\right)$corresponds to the charge displacement of electrons within the unit cell, and $\eta_{y}^{\pm}\left(k\right)$ indicates the dipole moment displacement, the phase (polarization) $\mu_{x}^{\pm}\left(k\right)$ of $\tilde{W}_{x,k}^{\pm}$ should represent the quadrupole moment displacement with respect to the unit cell center, which implies the existence of an octupole moment. This can be verified from the two bands $\mu_{x}^{\pm}\left(k\right)$ in Fig. S5d, where they touch at *1*/2 for *q_y_*=±*π*, and shift to 0.1(0.9) for *q_y_*=0, signifying the displacement of the center of quadrupole moment. While for the trivial case with *λ*=1/3.3 (Supplementary Fig. S6), both bands of $\mu_{x}^{\pm}\left(k\right)$ are close to 0 (2*π*) for all *q_y_* and *q_x_*, indicating the absence of quadrupole moment displacement, that is, the absence of octupole moment. Note that the topology of octupole moment is only determined by the value of *λ* and is not dependent on the order of the nested Wilson loop along *q_x_*, *q_y_*, and *q_z_*. This is a clear evidence that the octupole corner mode is protected by the topology of the bulk (lattice symmetry) instead of a trivial edge property. Detailed calculation procedure of the octupole moment is given in method.

Here we show the detailed process to determine the nontrivial octupole moment of our 3D topological circuit. Firstly, we should note that although the circuit Laplacian in Eq. (2) does not give directly the eigenfrequency of the circuit because itself is dependent on frequency, it can be used to analyze the topological invariant and symmetries of the topological circuit, when the inductive and capacitive components in the diagonal terms are cancelled out at $\omega\text{=}\omega_{0}\text{=}1/\sqrt{C_{1}L_{1}}$. It is proved that the topological invariant of the circuit calculated from both the circuit Laplacian and circuit Hamiltonian are identical [37]. Hence, we will use the circuit Laplacian at *ω_0_* given in Eq. (2) as the starting point in the calculation procedure of the nested Wilson loops, which mainly involves three steps:

**Step 1**: Calculate the Wilson loop along *q_z_*,

$W_{z,k}\text{=}F_{q+2\pi-\Delta q_{z}}F_{q+2\pi-2\Delta q_{z}}\cdots F_{q+\Delta q_{z}}F_{q}$ (S21)

using the Wilson line element

$\left[F_{q}\right]^{mn}=\left\langle u_{q}^{m}\left|\left.u_{q}^{n}\right\rangle\right.\right.$ (S22)

where $\left|\left.u_{q}^{m}\right\rangle\right.$ is the four degenerate eigenstates (*m,n*=1,2,3,4) of $J_{\lambda}\left(\omega_{0},q\right)$ with positive eigenvalues. $\Delta_{q_{i}}=\frac{2\pi}{N_{i}}$, with *N*_i_ the lattice number along the *i*-axis (*i=x,y,z*). Here, the first subscript *z* of $W_{z,q}$ indicates that the Wilson loop is calculated along *q_z_*. Note that analytical solutions of $\left|\left.u_{q}^{n}\right\rangle\right.$ are used for the calculation of the 4×4 non-abelian Wilson loop $W_{z,q}$ to guarantee correct orders among the four degenerate eigenstates (see details in Supplemental Materials Note S4). This Wilson loop has an associate Hamiltonian $H_{surface}\left(q\right)$ given by,

$W_{z,q}\text{=}e^{-iH_{surface}\left(q\right)}$ (S23)

which has the same topology of the Hamiltonian at the surface of the 3D circuit in the *xy* plane, and is related to the Hamiltonian of the 2D circuit with quadrupole moment. Now we can obtain the eigenvalue $e^{i2\pi\nu_{z}^{j}\left(q\right)}$ and eigenstate  of $W_{z,q}$,

$W_{z,q}^{\pm}|\nu_{z,q}^{\pm\text{,}j}\rangle=e^{i2\pi\nu_{z}^{\pm\text{, }j}\left(q\right)}|\nu_{z,q}^{\pm,j}\rangle$ (S24)

where the four eigenstates are gapped with two two-fold degenerate Wannier bands  (*j*=1,2), as shown in Fig. S5b.

**Step 2**: Further probe the sector and characterize its topology by constructing the nested Wilson loop along *q_y_*

$\begin{matrix} \left[\tilde{W}^{\text{+}}_{y,q}\right]^{j,j'}\text{=}\langle w_{z,q+N_{y}\Delta_{qy}}^{+,j}|\left.w_{z,q+\left(N_{y}-1\right)\Delta_{qy}}^{+,\mathrm{r}}\right\rangle\langle w_{z,q+\left(N_{y}-1\right)\Delta_{qy}}^{+,\mathrm{r}}|\cdots\\ \cdots\left.\left|w_{z,q+\Delta_{qy}}^{+,\mathrm{s}}\right.\right\rangle\langle w_{z,q+\Delta_{qy}}^{+,\mathrm{s}}|\left.w_{z,q}^{+,\mathrm{j}'}\right\rangle\end{matrix}$ (S25)

in which the Wannier states $|w_{z,q}^{+,j}\rangle$is obtained from the linear combination of the eigenstates of $\left|\left.u_{q}^{n}\right\rangle\right.$ using the sub Wannier bands obtained in step 1,

$|w_{z,q}^{+,j}\rangle\text{=}\sum_{n=1}^{N_{occ}} |u_{q}^{n}\rangle\left[\nu_{z,q}^{+,j}\right]^{n}$ (S26)

in which$j,r,\ldots s,j'=1,2$. Note that the 1^st^-round Wilson loop $W_{z,q}$ happens to be diagonal for all *q_x_* and *q_y_*, which indicates that the Wannier bands takes the form of identity matrix. Hence, the Wannier states $|w_{z,q}^{+,j}\rangle$ are simply the first and second bands of $\left|\left.u_{q}^{n}\right\rangle\right.$ (*n*=1,2) (see details in Supplementary Note S4). $W_{y,q}$ has an associated Hamiltonian $H_{hinge}\left(k\right)$ given by,

$W_{y,q}\text{=}e^{-iH_{hinge}\left(k\right)}$ (S27)

which shares the same topological properties with the Hamiltonian at the one-dimensional boundaries of the two-dimensional *xy* plane surface of the 3D circuit. Similar to step 1, we can characterize the topology at the one-dimensional hinge by diagonalizing the Wilson loop $W_{y,q}$,

$W_{y,q}|\eta_{y,q}^{\pm}\rangle=e^{i2\pi\eta_{y}^{\pm}\left(q\right)}|\eta_{y,q}^{\pm}\rangle$ (S28)

The hinge Hamiltonian $H_{hinge}\left(k\right)$ is gapped due to the gapped Wannier bands $\eta_{y}^{\pm}\left(q\right)$, with each band being a non-degenerate single band.

**Step 3:** To further probe into the topology of the one-dimensional TI at the hinge of the 3D circuit, we once again define two Wannier sectors

$|w_{y,q}^{\pm}\rangle\text{=}\sum_{n=1}^{N_{occ}} |u_{q}^{n}\rangle\left[\eta_{y,q}^{\pm}\right]^{n}$ (S29)

and then calculate the nested Wilson loop $\tilde{W}_{x,q}$along *k_x_* using the $\eta_{y}^{\text{+}}$ sector

$\begin{matrix} \tilde{W}_{x,q}^{+}\text{=}\langle w_{y,q+N_{x}\Delta_{qx}}^{+}|\left.w_{y,q+\left(N_{x}\text{-}1\right)\Delta_{qx}}^{+}\right\rangle\langle w_{y,q+\left(N_{x}\text{-}1\right)\Delta_{qx}}^{+}|\cdots\\ \cdots\left.w_{y,q+\Delta_{qx}}^{+}\right\rangle\langle w_{y,q+\Delta_{qx}}^{+}|\left.w_{y,q}^{+}\right\rangle\end{matrix}$ (S30)

Now, the topology of the octupole moment can be determined by inspecting whether the positive and negative polarization bands  of the 3^rd^-round Wilson loop touch at 1/2 or 0, which are the topological invariant for the nontrivial and trivial cases, respectively. A nontrivial polarization of 1/2 implies a dipole moment in the Hinge Hamiltonian, and a quadrupole moment in the surface Hamiltonian, and thus guaranteeing an octupole moment for the entire Hamiltonian of the 3D circuit. Note that the negative polarization (blue) in Fig. S5d is obtained by choosing the negative bands in all three nested Wilson loops.

**Analytical solutions for the eigenstates for the calculation of non-abelian Wilson loops:** One should notice that in the calculation of the Wannier bands, the first two Wilson loops are 4×4 and 2×2 non-abelian Wilson loops. As we need to access each eigenvalue and eigenstate of these non-abelian Wilson loops, but not simply their products that are the non-abelian berry phase, a correct order among these degenerate states must be guaranteed during the successive multiplication of each Wilson element. Here, we solve this issue by employing the analytical solutions of the eigenstates for the 8×8 circuit Laplacian $J_{\lambda}\left(\omega_{0},q\right)$, which are recalled here as,

$J_{\lambda}\left(\omega_{0},q\right)=i\sqrt{\frac{C_{1}}{L_{1}}}\left[\lambda\sin q_{y}\Gamma'{}_{1}+\left(1+\lambda\cos q_{y}\right)\Gamma'{}_{2}+\lambda\sin q_{x}\Gamma'{}_{3}+\left(1+\lambda\cos q_{x}\right)\Gamma'{}_{4}+\lambda\sin q_{z}\Gamma'{}_{5}+\left(1+\lambda\cos q_{z}\right)\Gamma'{}_{6}\right]$ (S31)

where $\Gamma'_{0}=\xi_{3}\otimes\Gamma_{0}$,$\Gamma'_{1}=\xi_{3}\otimes\Gamma_{1}$,$\Gamma'_{2}=\xi_{3}\otimes\Gamma_{2}$,$\Gamma'_{3}=\xi_{3}\otimes\Gamma_{3}$,$\Gamma'_{4}=\xi_{3}\otimes\Gamma_{4}$,$\Gamma'_{5}=\xi_{2}\otimes\text{I}_{4\times4}$,$\Gamma'_{6}=i\Gamma'_{0}\Gamma'_{1}\Gamma'_{2}\Gamma'_{3}\Gamma'_{4}\Gamma'_{5}$, and $\Gamma_{1}=\tau_{2}\otimes\sigma_{1}$,$\Gamma_{2}=\tau_{2}\otimes\sigma_{2}$,$\Gamma_{3}=\tau_{2}\otimes\sigma_{3}$,$\Gamma_{4}=\tau_{1}\otimes\sigma_{0}$, *τ_v_, σ_v_, ζ_v_* are Pauli matrices corresponding to the internal degrees of freedom within a unit cell. For simplicity, Eq. (S31) is rewrite as,

$H=\left[\begin{matrix} & & B & A & C & & & \\ & & -\bar{A} & \bar{B} & & C & & \\ \bar{B} & -A & & & & & C & \\ \bar{A} & B & & & & & & C \\ \bar{C} & & & & & & -B & -A \\ & \bar{C} & & & & & \bar{A} & -\bar{B} \\ & & \bar{C} & & -\bar{B} & A & & \\ & & & \bar{C} & -\bar{A} & -B & & \end{matrix}\right]$ (S32)

Where a

(S33)

Eq.(31) has two four-fold degenerate eigenvalues

$\pm E=\pm\sqrt{\left|A\right|^{2}+\left|B\right|^{2}+\left|C\right|^{2}}=\pm\sqrt{3+3\lambda^{2}+2\lambda\left(\cos q_{x}+\cos q_{y}+\cos q_{z}\right)}$ (S34)

and degenerate eigenstates corresponding to positive eigenvalues,

$V_{+}\left(H\right)=\left[\begin{matrix} A & B & 0 & E \\ \bar{B} & -\bar{A} & E & 0 \\ 0 & E & -A & \bar{B} \\ E & 0 & B & \bar{A} \\ 0 & 0 & 0 & \bar{C} \\ 0 & 0 & \bar{C} & 0 \\ 0 & \bar{C} & 0 & 0 \\ \bar{C} & 0 & 0 & 0 \end{matrix}\right]$ (S35)

Now we need to orthogonalize the four degenerate states in Eq.(S23) before it can be sent for calculating the 1^st^ round Wilson loop. The subspace spanned by $V_{+}\left(H\right)$ is,

$V_{+}\left(H\right)^{\dagger}V_{+}\left(H\right)=2E\left[\begin{matrix} E & 0 & B & \bar{A} \\ 0 & E & -A & \bar{B} \\ \bar{B} & -\bar{A} & E & 0 \\ A & B & 0 & E \end{matrix}\right]$ (S36)

From which we can construct a new 4×4 Hamiltonian as,

$HH=\left[\begin{matrix} 0 & 0 & B & \bar{A} \\ 0 & 0 & -A & \bar{B} \\ \bar{B} & -\bar{A} & 0 & 0 \\ A & B & 0 & 0 \end{matrix}\right]$ (S37)

Note that Eq. (S37) is exactly the Hamiltonian of the 2D case with quadrupole moment [2], having two two-fold degenerate eigenvalues

$\pm D=\pm\sqrt{\left|A\right|^{2}+\left|B\right|^{2}}=\pm\sqrt{2+2\lambda^{2}+2\lambda\left(\cos q_{x}+\cos q_{y}\right)}$ (S38)

and eigenstates,

$V\left(HH\right)=\left[\begin{matrix} -\bar{A}-B & -\bar{A}+B & \bar{A}+B & \bar{A}-B \\ A-\bar{B} & -A-\bar{B} & -A+\bar{B} & A+\bar{B} \\ D & -D & D & -D \\ D & D & D & D \end{matrix}\right]$ (S39)

Now $V_{+}\left(H\right)$ can be orthogonalized by

$V=V_{+}\left(H\right)V\left(HH\right)=\left[\begin{matrix} D\left(E-D\right) & D\left(E-D\right) & D\left(E+D\right) & D\left(E+D\right) \\ D\left(E-D\right) & -D\left(E-D\right) & D\left(E+D\right) & -D\left(E+D\right) \\ \left(A-\bar{B}\right)\left(E-D\right) & -\left(A+\bar{B}\right)\left(E-D\right) & -\left(A-\bar{B}\right)\left(E+D\right) & \left(A+\bar{B}\right)\left(E+D\right) \\ -\left(\bar{A}+B\right)\left(E-D\right) & -\left(\bar{A}-B\right)\left(E-D\right) & \left(\bar{A}+B\right)\left(E+D\right) & \left(\bar{A}-B\right)\left(E+D\right) \\ \bar{C}D & \bar{C}D & \bar{C}D & \bar{C}D \\ \bar{C}D & -\bar{C}D & \bar{C}D & -\bar{C}D \\ \left(A-\bar{B}\right)\bar{C} & -\left(A+\bar{B}\right)\bar{C} & -\left(A-\bar{B}\right)\bar{C} & \left(A+\bar{B}\right)\bar{C} \\ -\left(\bar{A}+B\right)\bar{C} & -\left(\bar{A}-B\right)\bar{C} & \left(\bar{A}+B\right)\bar{C} & \left(\bar{A}-B\right)\bar{C} \end{matrix}\right]$ (S40)

and after normalization,

$\tilde{V}=\frac{1}{\sqrt{8D^{2}E}}F\text{=}\frac{1}{\sqrt{8D^{2}E}}\left[\begin{matrix} D\sqrt{E-D} & D\sqrt{E-D} & D\sqrt{E+D} & D\sqrt{E+D} \\ D\sqrt{E-D} & -D\sqrt{E-D} & D\sqrt{E+D} & -D\sqrt{E+D} \\ \left(A-\bar{B}\right)\sqrt{E-D} & -\left(A+\bar{B}\right)\sqrt{E-D} & -\left(A-\bar{B}\right)\sqrt{E+D} & \left(A+\bar{B}\right)\sqrt{E+D} \\ -\left(\bar{A}+B\right)\sqrt{E-D} & -\left(\bar{A}-B\right)\sqrt{E-D} & \left(\bar{A}+B\right)\sqrt{E+D} & \left(\bar{A}-B\right)\sqrt{E+D} \\ \bar{C}D/\sqrt{E-D} & \bar{C}D/\sqrt{E-D} & \bar{C}D/\sqrt{E+D} & \bar{C}D/\sqrt{E+D} \\ \bar{C}D/\sqrt{E-D} & -\bar{C}D/\sqrt{E-D} & \bar{C}D/\sqrt{E+D} & -\bar{C}D/\sqrt{E+D} \\ \left(A-\bar{B}\right)\bar{C}/\sqrt{E-D} & -\left(A+\bar{B}\right)\bar{C}/\sqrt{E-D} & -\left(A-\bar{B}\right)\bar{C}/\sqrt{E+D} & \left(A+\bar{B}\right)\bar{C}/\sqrt{E+D} \\ -\left(\bar{A}+B\right)\bar{C}/\sqrt{E-D} & -\left(\bar{A}-B\right)\bar{C}/\sqrt{E-D} & \left(\bar{A}+B\right)\bar{C}/\sqrt{E+D} & \left(\bar{A}-B\right)\bar{C}/\sqrt{E+D} \end{matrix}\right]$ (S41)

The fact that the 1^st^-round Wilson loop $W_{z,k}\text{=}F_{k+2\pi-\Delta k_{z}}F_{k+2\pi-2\Delta k_{z}}\cdots F_{k+\Delta k_{z}}F_{k}$ is a diagonal matrix can be found by calculating,

$\tilde{V}^{\dagger}\partial_{q_{z}}\tilde{V}=\frac{-i\lambda\cos q_{z}-i\lambda^{2}}{2E}\left[\begin{matrix} \frac{1}{E-D} & & & \\ & \frac{1}{E-D} & & \\ & & \frac{1}{E+D} & \\ & & & \frac{1}{E+D} \end{matrix}\right]$ (S42)

and integrating it along *q_z_*,

$\int\tilde{V}^{\dagger}\partial_{q_{z}}\tilde{V}dq_{z}=\int\frac{-i\lambda\cos q_{z}-i\lambda^{2}}{2E}\left[\begin{matrix} \frac{1}{E-D} & & & \\ & \frac{1}{E-D} & & \\ & & \frac{1}{E+D} & \\ & & & \frac{1}{E+D} \end{matrix}\right]dq_{z}$ (S43)

Note that in the numerical calculation of the non-abelian Wilson loop, one should consider applying singular value decomposition to each Wilson element to guarantee its unitary property.


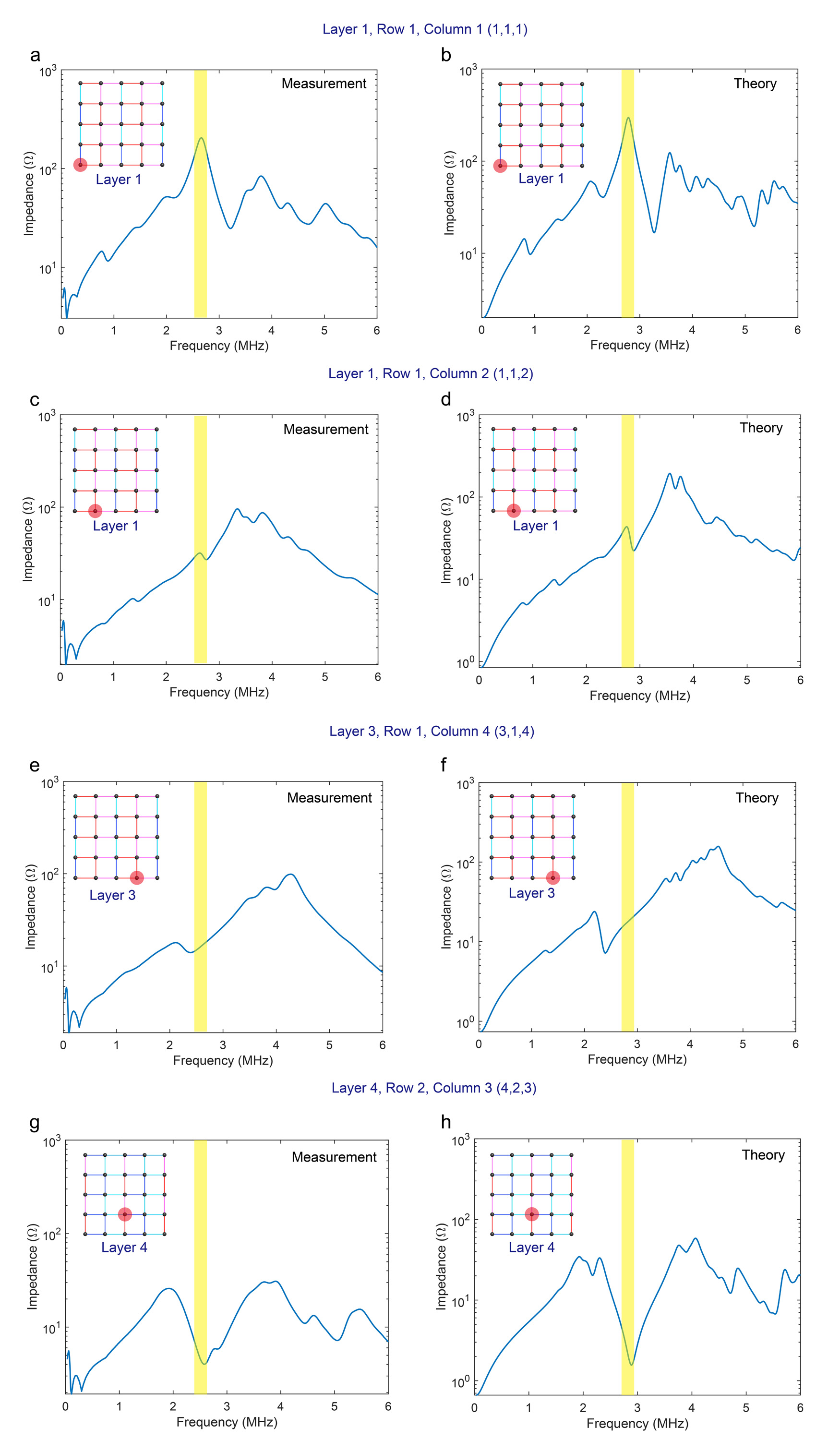


**Supplementary Fig. S1 Theoretically calculated impedance spectra at four chosen nodes.** **(a)** Layer 1, row 1, column 1. **(b)** Layer 1, row 1, column 2. **(c)** Layer 3, row 1, column 4. **(d)** Layer 4, row 2, column 3. Yellow region indicate the bandgap where the corner state locates. Note the two-point impedance *Z_ab_* is measured across the current node and the next nearest node along the *x*-direction.


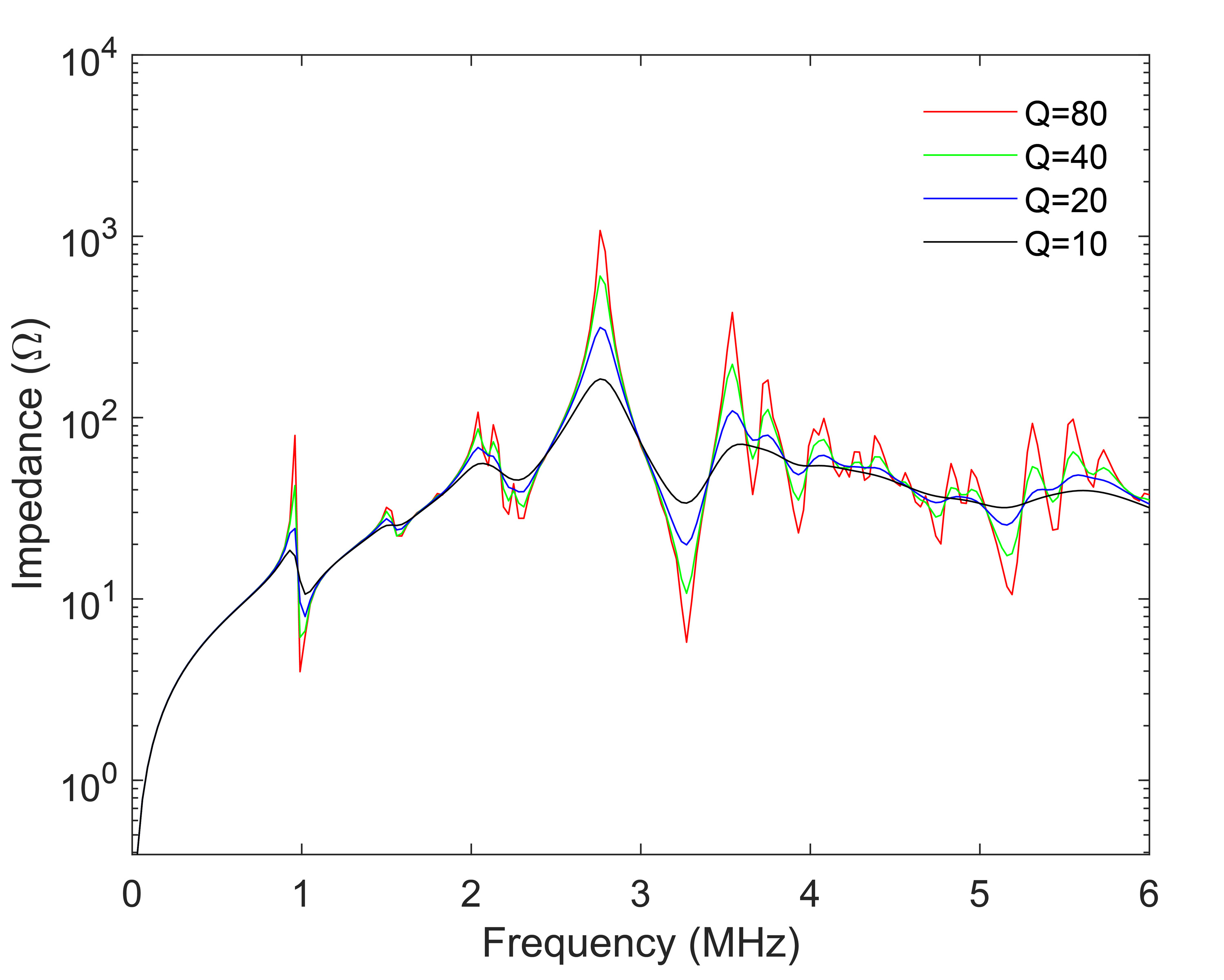


**Supplementary Fig. S2 Calculated impedance spectra at corner A with Q-factor ranging from 10 to 80.**


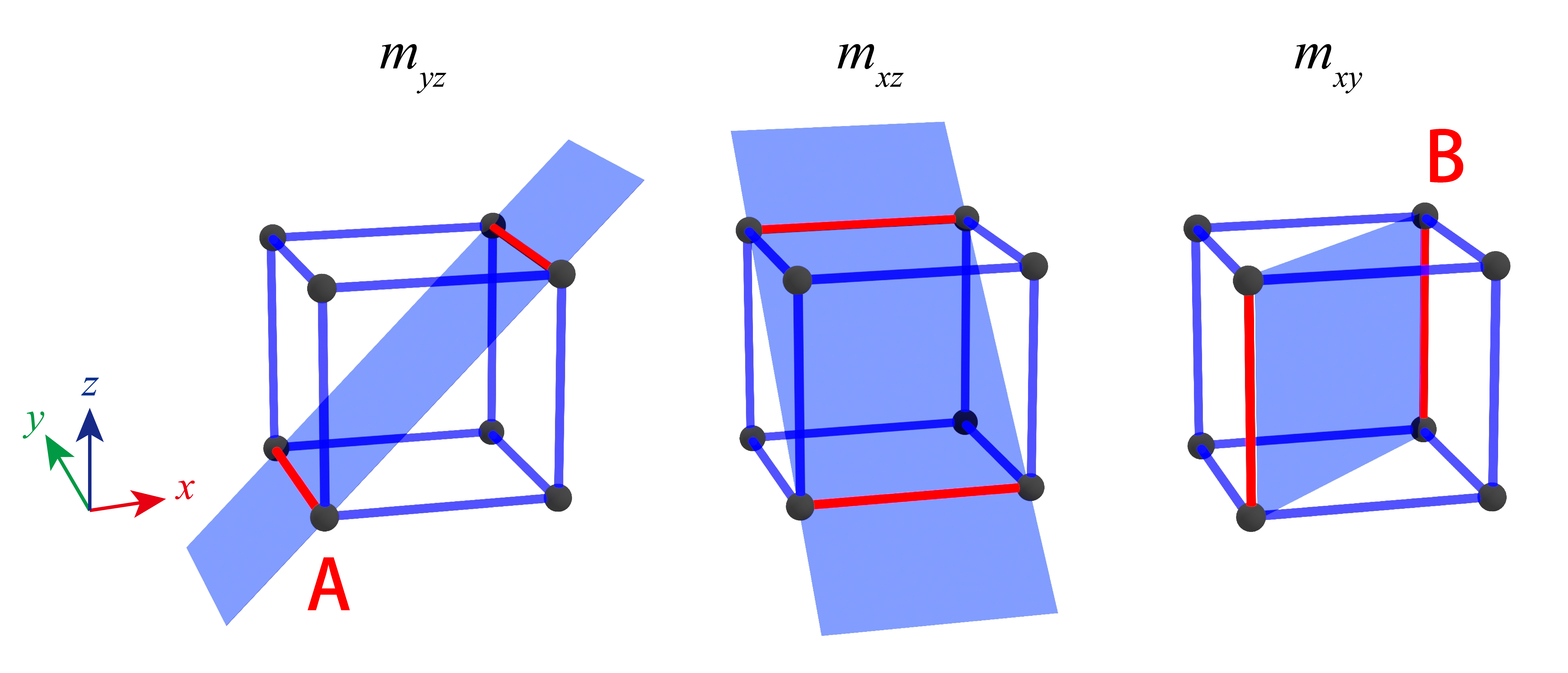


**Supplementary Fig. S3 Schematic illustration of the three mirror symmetry $\hat{m}_{xz}$*,* $\hat{m}_{yz}$*,* and $\hat{m}_{xy}$in the finite circuit.** **Corner states are potentially protected by the three mirror symmetries in the two corners labeled as A and B.**


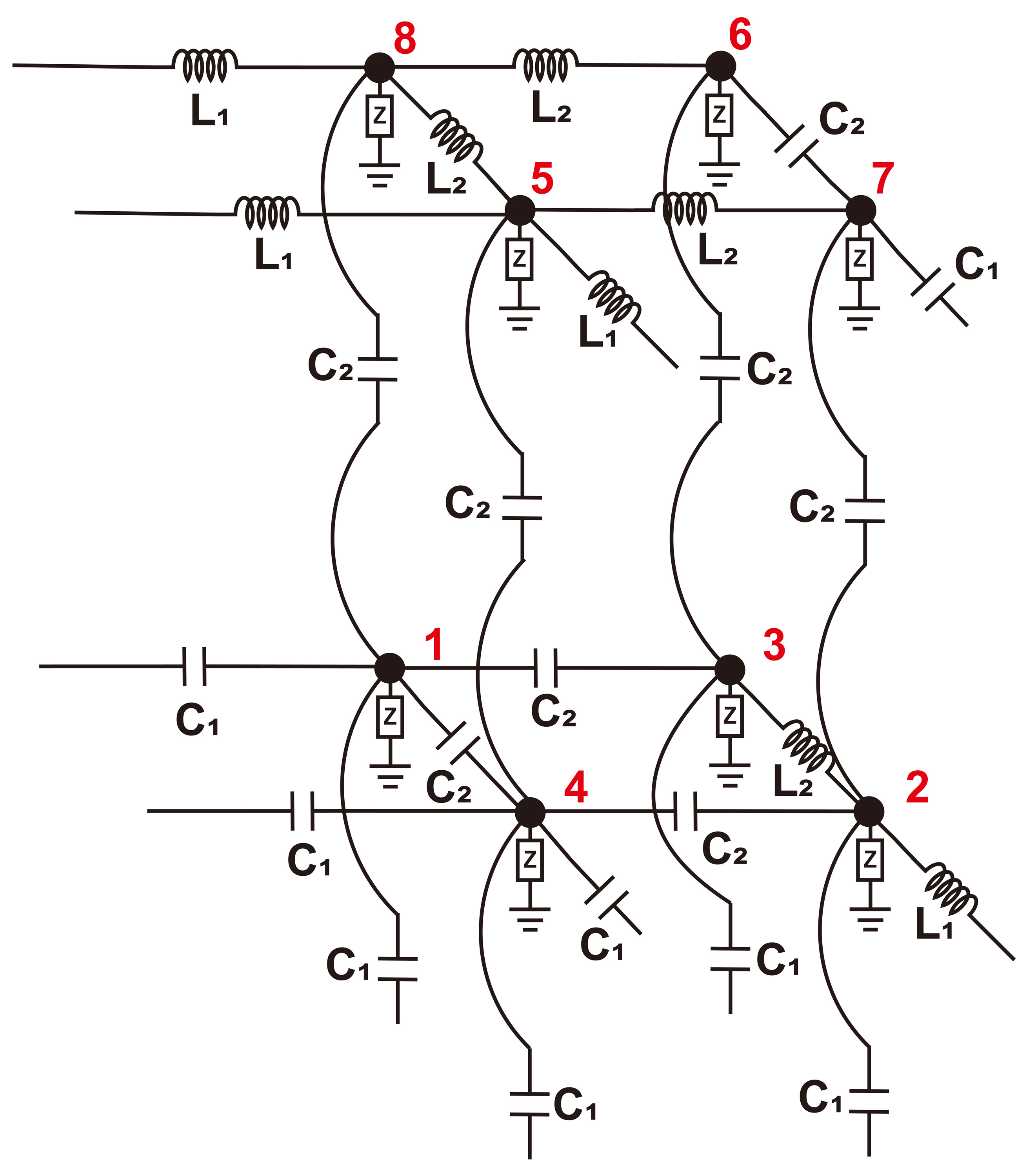


**Supplementary Fig. S4 Type-Ⅱ unit cell of the circuit**


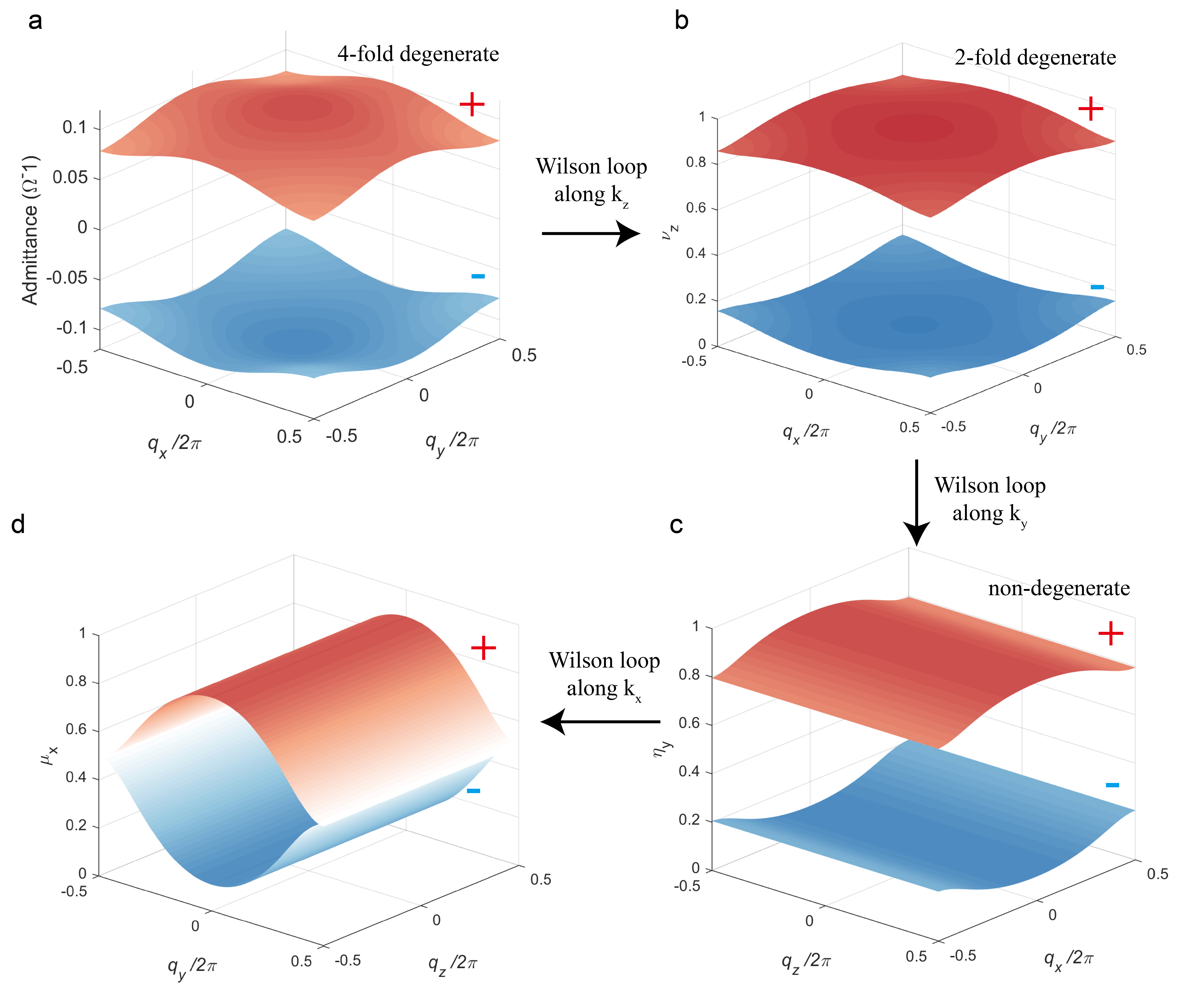


**Fig. S5 Process to determine the topology of the octupole moment of the bulk circuit with** ***λ*=3.3 through a three-step nested Wilson loops. (a)** Eigenvalue spectra of the circuit Laplacian at *ω_0_* for *q_z_*=-0.6π. Each band is four-fold degenerate. **(b)** Phase of eigenvalue $\nu_{y}^{j,\pm}\left(q\right)$obtained from the 1^st^-round Wilson loop along *q_z_*. Each Wannier band is two-fold degenerate. **(c)** Phase of eigenvalue $\eta_{y}^{\pm}\left(q\right)$obtained from the 2^st^-round Wilson loop along *q_y_*. The two Wannier bands are separated. **(d)** Polarization $\mu_{x}^{\pm}\left(q\right)$ obtained from the phase of the 3^rd^-round Wilson loop along *q_x_.* The lower band (blue) is obtained by choosing the negative bands in all the three-step nested Wilson loops.


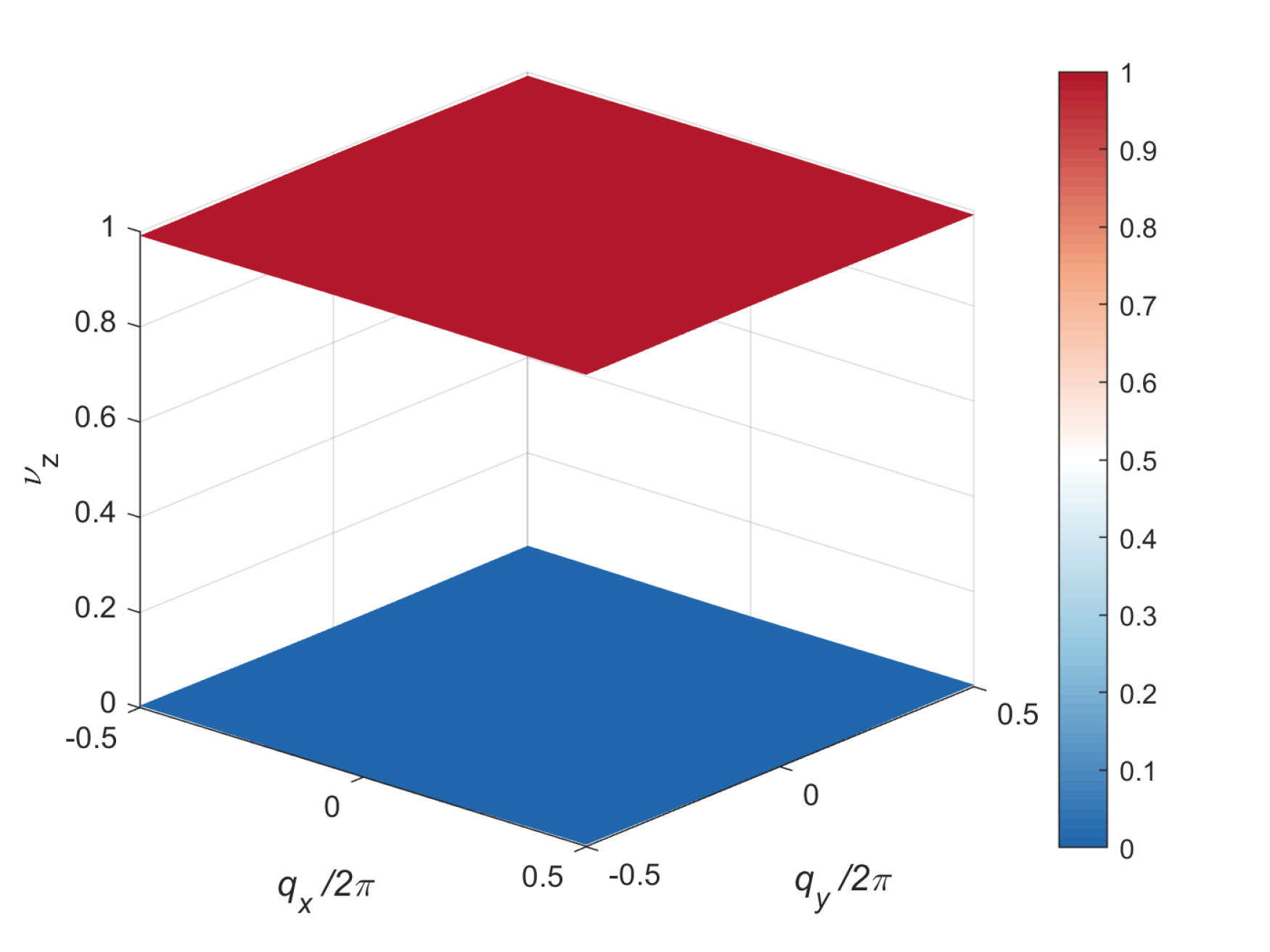


**Supplementary Fig. S6** Polarization $\mu_{x}^{\pm}\left(q\right)$ obtained from the phase of the 3^rd^-round Wilson loop along *q_x_* for *λ*=1/3.3 Both bands are close to 0 and 2*π* for all *q_y_* and *q_x_*, indicating the absence of quadrupole moment displacement, that is the absence of octupole moment.


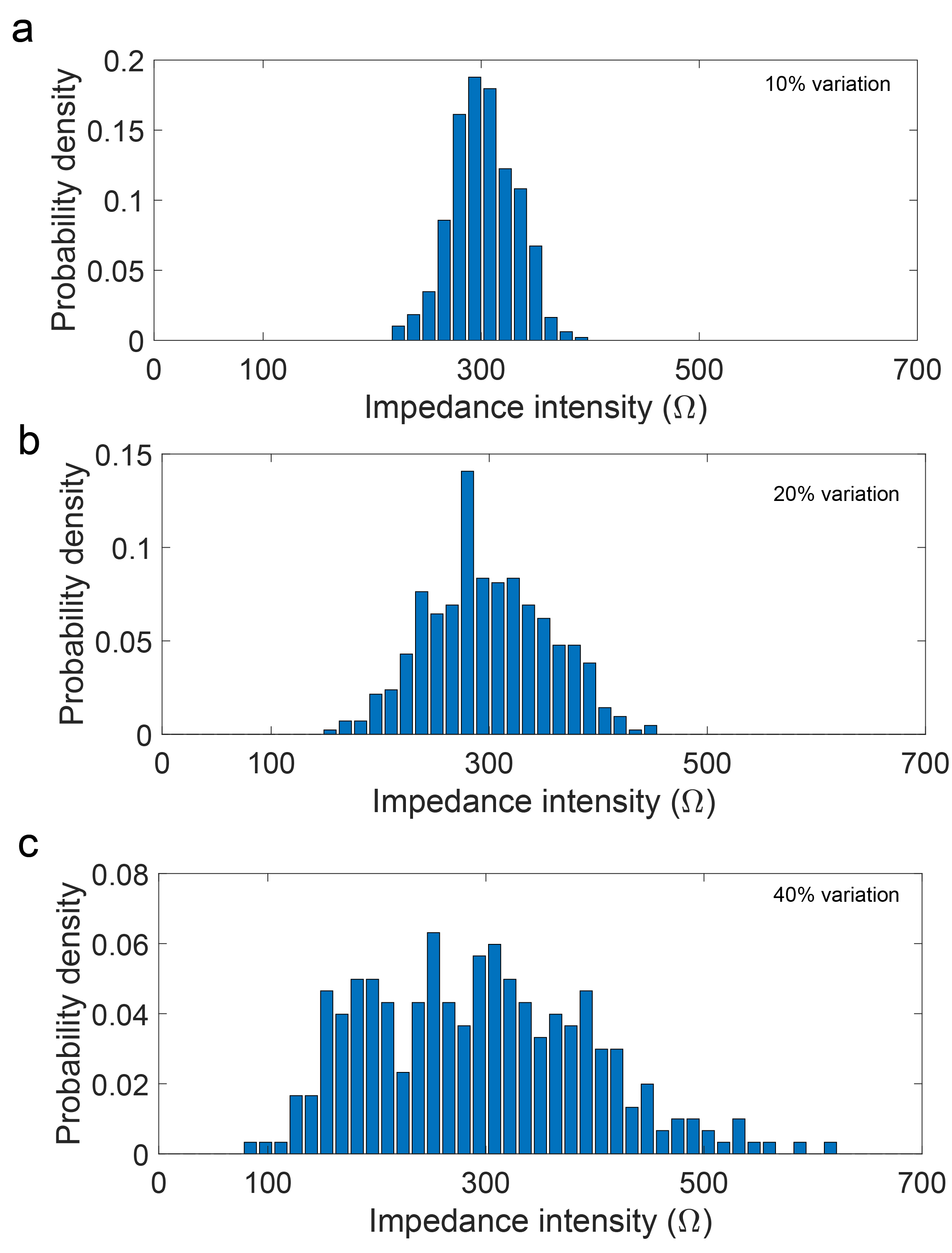


**Supplementary Fig. S7 (a-c) Probability distribution of the corner state intensity with 10%, 20%, 40% circuit component variations, respectively.**

**Table. S1 Grounded terms for the unit cell circuit in Fig. 1b**

| Node index | Grounded term | Node index | Grounded term |
| --- | --- | --- | --- |
| 1 | L_1g_=L_1_/(3+3λ) | 5 | C_1g_=C_1_+C_2_ |
| 2 | L_2g_=L_1_/(1+λ) | 6 | L_2g_=L_1_/(1+λ) |
| 3 | L_2g_=L_1_/(1+λ) | 7 | L_2g_=L_1_/(1+λ) |
| 4 | L_1g_= L_1_/(3+3λ) | 8 | C_1g_=C_1_+C_2_ |

**Table. S2 Grounded terms for the finite circuit at layer 1**

|  | 1 | 2 | 3 | 4 | 5 |
| --- | --- | --- | --- | --- | --- |
| 5 | Lcorner_3=1μH (1μH) | Cx01_1=6.6nF (6.8nH) | Cx01_2=1nF (1nF)  Lx01_2=3.3μH (3.3μH) | Cx01_1=6.6nF (6.8nF) | Lcorner_4=3.3μH (3.3μH) |
| 4 | L_y00_=0.767μH (0.75μH) | Cxoy0_1=7.6nF (7.5nF) | Lxoy0_2=3.3μH (3.3μH) | Cxoy0_1=7.6nF (7.5nF) | L_y01_=1.65μH (1.62μH) |
| 3 | L_y00_=0.767μH (0.75μH) | Cxoy0_1=7.6nF (7.5nF) | Lxoy0_2=3.3μH (3.3μH) | Cxoy0_1=7.6nF (7.5nF) | Ly01=1.65μH (1.62μH) |
| 2 | L_y00_=0.767μH (0.75μH) | Cxoy0_1=7.6nF (7.5nF) | Lxoy0_2=3.3μH (3.3μH) | Cxoy0_1=7.6nF (7.5nF) | Lg_y01=1.65μH (1.62μH) |
| 1 | Lcorner_1=3.3μH (3.3μH) | Cx00_1=4.3nF (4.3 nF) | Cx00_2=2.3nF (2.3nF) | Cx00_1=4.3nF (4.3nF) | Ccorner_2=1.3nF (1.3nF) |

**Table. 3 Grounded terms for the finite circuit at layer 2**

|  | 1 | 2 | 3 | 4 | 5 |
| --- | --- | --- | --- | --- | --- |
| 5 | Lz10_1=1.65μH (1.62μH) | Lxoz1_1=0.277μH (0.27μH) | Lxoz1_2=0.623μH (0.62μH) | Lxoz1_1=0.277μH (0.27μH) | L_z11_=0.767μH (0.75μH) |
| 4 | Lyoz0_1=3.3μH (3.3μH) | L_1g_=0.256μH (0.27μH) | L_2g_=0.767μH (0.75μH) | L_1g_=0.256μH (0.27μH) | Lyoz1_1=1μH (1μH) |
| 3 | Lyoz0_1=3.3μH (3.3μH) | L_1g_=0.256μH (0.27μH) | L_2g_=0.767μH (0.75μH) | L_1g_=0.256μH (0.27μH) | Lyoz1_1=1μH (1μH) |
| 2 | Lyoz0_1=3.3μH (3.3μH) | L_1g_=0.256μH (0.27μH) | L_2g_=0.767μH (0.75μH) | L_1g_=0.256μH (0.27μH) | Lyoz1_1=1μH (1μH) |
| 1 | L_z00_=0.767μH (0.75μH) | Lxoz0_1=0.344μH (0.33μH) | Lxoz0_2=0.434μH (0.47μH) | Lxoz0_1=0.344μH (0.33μH) | Lz01_1=0.5μH (0.47μH) |

**Table. 4 Grounded terms for the finite circuit at layer 3**

|  | 1 | 2 | 3 | 4 | 5 |
| --- | --- | --- | --- | --- | --- |
| 5 | Lz10_2=0.5μH (0.47μH) | Cxoz1_3=3.3μH (3.3μH) | Lxoz1_4=1μH (1μH) | Cxoz1_3=3.3μH (3.3μH) | L_z11_=0.767μH (0.75μH) |
| 4 | Lyoz0_2=0.434μH (0.47μH) | C_1g_=4.3nF (4.3nF) | L_2g_=0.767μH (0.75μH) | C_1g_=4.3nF (4.3nF) | Lyoz1_2=0.623μH (0.62μH) |
| 3 | Lyoz0_2=0.434μH (0.47μH) | C_1g_=4.3nF (4.3nF) | L_2g_=0.767μH (0.75μH) | C_1g_=4.3nF (4.3nF) | Lyoz1_2=0.623μH (0.62μH) |
| 2 | Lyoz0_2=0.434μH (0.47μH) | C_1g_=4.3nF (4.3nF) | L_2g_=0.767μH (0.75μH) | C_1g_=4.3nF (4.3nF) | Lyoz1_2=0.623μH (0.62μH) |
| 1 | L_z00_=0.767μH (0.75μH) | Cxoz0_3=1nF (1nF) | Lxoz1_4=1μH (1μH) | Cxoz0_3=1nF (1nF) | Lz01_2=1.65μH (1.62μH) |

**Table. 5 Grounded terms for the finite circuit at layer 4**

|  | 1 | 2 | 3 | 4 | 5 |
| --- | --- | --- | --- | --- | --- |
| 5 | Lz10_1=1.65μH (1.62μH) | Lxoz1_1=0.277μH (0.27μH) | Lxoz1_2=0.623μH (0.62μH) | Lxoz1_1=0.277μH (0.27μH) | L_z11_=0.767μH (0.75μH) |
| 4 | Lyoz0_1=3.3μH (3.3μH) | L_1g_=0.256μH (0.27μH) | L_2g_=0.767μH (0.75μH) | L_1g_=0.256μH (0.27μH) | Lyoz1_1=1μH (1μH) |
| 3 | Lyoz0_1=3.3μH (3.3μH) | L_1g_=0.256μH (0.27μH) | L_2g_=0.767μH (0.75μH) | L_1g_=0.256μH (0.27μH) | Lyoz1_1=1μH (1μH) |
| 2 | Lyoz0_1=3.3μH (3.3μH) | L_1g_=0.256μH (0.27μH) | L_2g_=0.767μH (0.75μH) | L_1g_=0.256μH (0.27μH) | Lyoz1_1=1μH (1μH) |
| 1 | L_z00_=0.767μH (0.75μH) | Lxoz0_1=0.344μH (0.33μH) | Lxoz0_2=0.434μH (0.47μH) | Lxoz0_1=0.344μH (0.33μH) | Lz01_1=0.5μH (0.47μH) |

**Table. 6 Grounded terms for the finite circuit at layer 5**

|  | 1 | 2 | 3 | 4 | 5 |
| --- | --- | --- | --- | --- | --- |
| 5 | Lcorner_7=0.589μH (0.56μH) | Cx11_1=4.3nF (4.3nF) | Lx11_2=1.43μH (1.47μH) | Cx11_1=4.3nF (4.3nF) | Lcorner_8=1μH (1μH) |
| 4 | L_y10_=0.5μH (0.47μH) | Cxoy1_1=5.3nF (5.3nF) | Lxoy1_2=1μH (1μH) | Cxoy1_1=5.3nF (5.3nF) | L_y11_=0.767μH (0.75μH) |
| 3 | L_y10_=0.5μH (0.47μH) | Cxoy1_1=5.3nF (5.3nF) | Lxoy1_2=1μH (1μH) | Cxoy1_1=5.3nF (5.3nF) | L_y11_=0.767μH (0.75μH) |
| 2 | L_y10_=0.5μH (0.47μH) | Cxoy1_1=5.3nF (5.3nF) | Lxoy1_2=1μH (1μH) | Cxoy1_1=5.3nF (5.3nF) | L_y11_=0.767μH (0.75μH) |
| 1 | Lcorner_5=1μH (1μH) | Cx10_1=2nF (2nF) | Cx10_2=1nF (1nF)  Lx10_2=3.3μH (3.3μH) | Cx10_1=2nF (2nF) | Lcorner_6=3.3μH (3.3μH) |

**Reference:**

1. Hofmann, T., et al. Chiral voltage propagation in a self-calibrated topolectrical Chern circuit. arXiv:1809.08687 (2018).
2. Benalcazar, W.A., Bernevig, B. A., Hughes, T. L. Quantized electric multipole insulators. Science, 357, 6346 (2017)
